# Development of a portable on-site applicable metagenomic data generation workflow for enhanced pathogen and antimicrobial resistance surveillance

**DOI:** 10.1038/s41598-023-46771-z

**Published:** 2023-11-11

**Authors:** Bram Bloemen, Mathieu Gand, Kevin Vanneste, Kathleen Marchal, Nancy H. C. Roosens, Sigrid C. J. De Keersmaecker

**Affiliations:** 1 Transversal Activities in Applied Genomics, Sciensano, Rue Juliette Wytsman 14, 1050 Brussels, Belgium; 2 Department of Information Technology, IDLab, Ghent University, IMEC, 9052 Ghent, Belgium; 3 Department of Plant Biotechnology and Bioinformatics, Ghent University, 9052 Ghent, Belgium

**Keywords:** Genomics, Next-generation sequencing, Metagenomics, Microbiome, Antimicrobials, Applied microbiology, Bacteria, Environmental microbiology, Infectious-disease diagnostics, Microbial genetics, Pathogens, Policy and public health in microbiology

## Abstract

Rapid, accurate and comprehensive diagnostics are essential for outbreak prevention and pathogen surveillance. Real-time, on-site metagenomics on miniaturized devices, such as Oxford Nanopore Technologies MinION sequencing, could provide a promising approach. However, current sample preparation protocols often require substantial equipment and dedicated laboratories, limiting their use. In this study, we developed a rapid on-site applicable DNA extraction and library preparation approach for nanopore sequencing, using portable devices. The optimized method consists of a portable mechanical lysis approach followed by magnetic bead-based DNA purification and automated sequencing library preparation, and resulted in a throughput comparable to a current optimal, laboratory-based protocol using enzymatic digestion to lyse cells. By using spike-in reference communities, we compared the on-site method with other workflows, and demonstrated reliable taxonomic profiling, despite method-specific biases. We also demonstrated the added value of long-read sequencing by recovering reads containing full-length antimicrobial resistance genes, and attributing them to a host species based on the additional genomic information they contain. Our method may provide a rapid, widely-applicable approach for microbial detection and surveillance in a variety of on-site settings.

## Introduction

Rapid, accurate and comprehensive diagnostics are key to identify outbreaks, and allow for improved pathogen surveillance to prevent further spread. Current pathogen detection methods have limitations that can restrict their use to dedicated laboratories with trained personnel. Culture-based approaches can be time-consuming and depend on the ability to cultivate the microbial species in question, while targeted molecular assays frequently require specialized equipment and prior knowledge. On the other hand, current point-of-care tests often only detect a limited range of predetermined pathogens^1^. Furthermore, these methods provide limited additional information, or can fail to identify a pathogen altogether. In recent years, high-throughput next-generation sequencing technologies (NGS) have created the possibility to comprehensively analyze samples without culturing or prior knowledge, in an approach termed metagenomic sequencing (mNGS)^2,3^. However, current short-read NGS technologies require specialized laboratories, skilled personnel, and high capital investments, while not providing information in real-time, impeding it to become a point-of-care test. Additionally, short reads provide a fractured view on the DNA content, limiting subsequent taxonomic or functional analyses^4^. Recently, the development of portable, real-time, long-read sequencing by Oxford Nanopore Technologies (ONT) has paved the way for rapid and comprehensive on-site microbial diagnostics and surveillance.

Several prior studies have demonstrated the added value of portable nanopore sequencing for diagnostic purposes. For example, Marin et al. recently published an on-farm 16S rDNA amplicon sequencing method using the portable Bento Bio Pro for DNA extraction and library preparation in combination with ONT MinION sequencing. The method was able to reliably detect *Campylobacter* in caecal samples from infected chickens in less than five hours, compared to more than 72 h required with conventional methods^5^. Additionally, Marcolungo et al. developed an amplicon sequencing approach to identify quarantine plant pathogens with high sensitivity^6^. Others have used in-situ nanopore amplicon sequencing for biodiversity assessment in a variety of remote settings^7–9^. Although these amplicon-based approaches have proven successful in terms of sensitivity and swiftness, they are targeted methods which deliver limited additional information, and in the previously mentioned examples were used to identify a limited set of pathogens or target genes.

In contrast, shotgun mNGS allows for a more comprehensive analysis by sampling all genetic material instead of a limited range of marker genes. Furthermore, it can provide additional functional insights by detecting (full length) virulence or antimicrobial resistance genes (ARG) and attributing them to a microbial species, while increasing taxonomic resolution^4,10–12^. In clinical settings, mNGS has already demonstrated added value by increased pathogen identification in lower respiratory tract, urinary and bloodstream infections when compared to culture-based or conventional tests^13–17^. Other examples of clinical mNGS applications include the earliest published SARS-CoV-2 genomes by Zhu et al., or rapid detection of bacterial pathogens and their antimicrobial resistance by Serpa et al.^18,19^. Apart from clinical diagnostics, mNGS has proven to be a capable tool to identify, characterize and trace the source of pathogens in agricultural, environmental and food safety contexts^20–23^. Applying mNGS on-site could be invaluable to further decrease sample-to-detection times, to increase the comprehensiveness of microbial detection methods, and to allow for more wide-spread use. Currently, such on-site mNGS experiments have mainly been performed in various remote locations using a combination of portable laboratory equipment and the ONT MinION sequencing device^24–27^. In these cases, sample transportation and preservation were not trivial, or were shown to impact taxonomic composition, highlighting the benefits of in-situ sample processing^27^. However, no controls or standardized samples were used in these studies, hindering evaluation of their performance and comparison with other methods^28^.

Even though nanopore sequencing itself can be performed on miniaturized devices, most current DNA extraction, library preparation, and data analysis methods still require non-portable devices and stable internet and electrical grid access, limiting potential use-cases. In recent years, several devices and methods have been developed to enable on-site metagenomic data generation. Examples include the Bento Bio^5^, SuperFastPrep-2^25^ and the Claremont Bio OmnilyseX and DNAexpress kits^29–31^. Additionally, the laptop-powered ONT VolTRAX allows for automated library preparation on a portable device. In this study we focused on the wet-lab aspect, and developed a method for portable on-site applicable mNGS by combining the Claremont Bio OmnilyseX, Bento Bio Pro and VolTRAX devices. When combined with potential future on-site bioinformatic analysis, this would provide a new tool for rapid in-situ diagnostics and surveillance. To optimize the data generation method, we used chicken fecal samples as a test-case, as the poultry gut microbiome has been demonstrated to contain a diverse repertoire of ARGs^32^. Additionally, chicken feces are known to be inhibitory towards molecular applications, making it a challenging proof-of-concept sample^33,34^. Starting from a previously published protocol^31^, we developed gradual improvements to arrive to a final wet-lab protocol that generated high quality nanopore mNGS data and can be used outside of laboratories. Using spike-in defined mock communities (DMC), we compared taxonomic classification and resistome profiling performance to other sequencing workflows, including a laboratory-based protocol.

## Results

### Optimization of a rapid metagenomic DNA extraction method, applicable on-site

**Figure 1:**
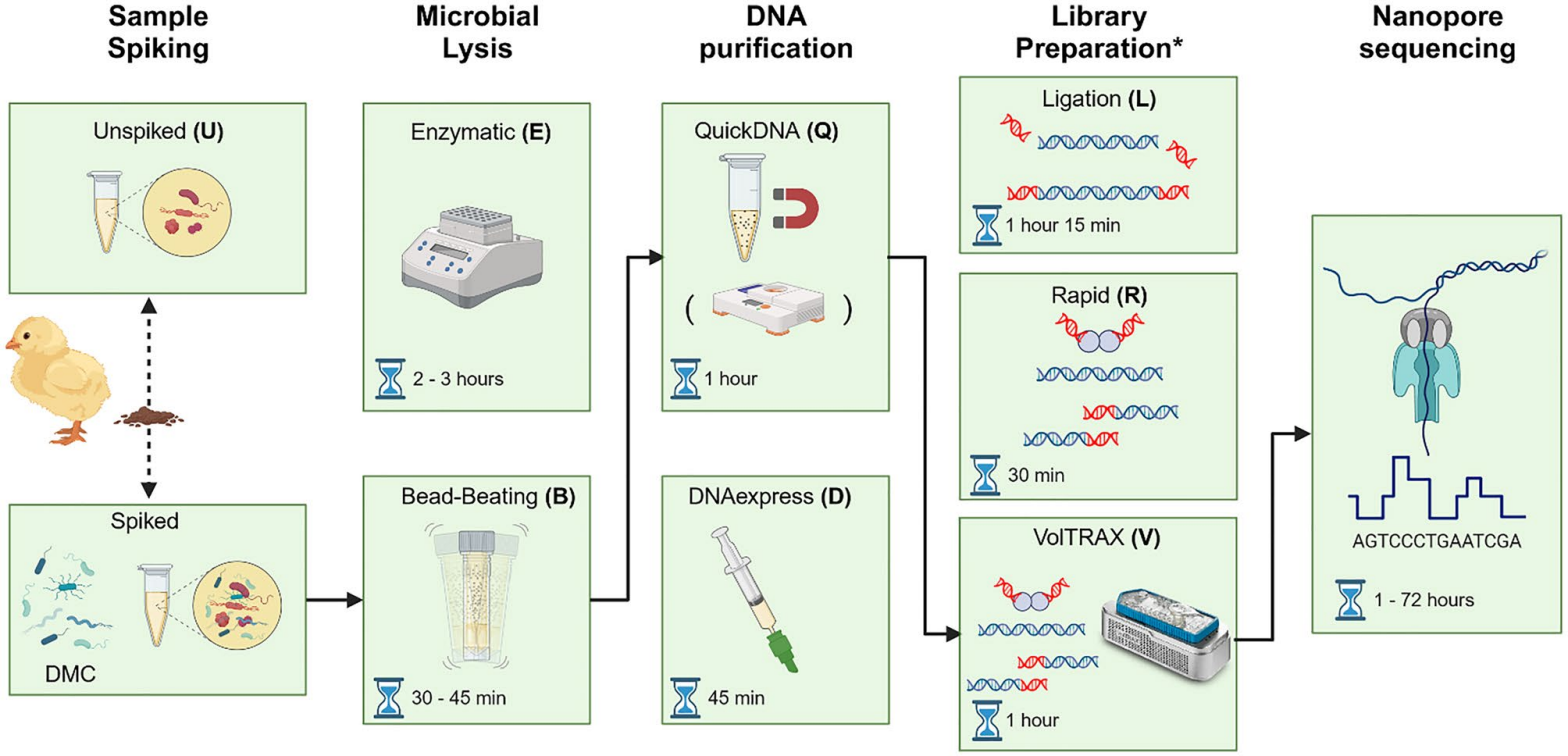
Overview of metagenomic workflows and abbreviations used, by step: sample spiking (spiked or unspiked (U)), microbial lysis (enzymatic (E) or bead-beating (B)), DNA purification (Quick-DNA magbead HMW (Q) or DNAexpress (D)) and library preparation (Ligation (L), Rapid (R) or VolTRAX (V)). As an example, the final, optimized on-site BQV workflow is indicated with arrows, starting from a spiked sample lysed with method B, purified with method Q and library preparation with method V. The following other workflows were assessed for sequencing performance: *UEQL* unspiked–enzymatic lysis–quick-DNA magbead HMW–ligation sequencing, *EQL* enzymatic lysis–quick-DNA magbead HMW–ligation sequencing, *EQR* enzymatic lysis–quick-DNA magbead HMW–rapid sequencing, *BDR* bead-beating–DNAexpress–rapid sequencing, *BQR* bead-beating–quick-DNA HMW magbead–rapid sequencing, *BQV* bead-beating–quick-DNA HMW magbead–voltrax sequencing. Hourglasses indicate the estimated time to carry out each step. *Before each library preparation method, additional purification was carried out, requiring approximately 15 min.

To reduce equipment requirements and sample processing times, the Claremont Bio DNAexpress kit was chosen to develop an on-site DNA extraction workflow^31^. The method consists of homogenizing and lysing the sample in a battery-powered Omnilyse X bead-beating tube (B) containing beads and lysis buffer, followed by the DNAexpress (D) column-based DNA purification protocol (Fig. 1). Various modifications were made to the protocol (abbreviated to BD, Table 1), and statistically compared to each other (supplementary Table S1). The protocol was initially followed as per manufacturer’s instructions, with varying bead-beating durations. However, BD yielded DNA of poor purity, with 260 nm/230 nm absorbance ratios (A260/230) being around 0.80 (Table 1), and no significant differences between bead-beating durations (p > 0.05, supplementary Table S1). Next, additional cleanup with AMPure XP beads was tested to improve DNA purity. Three strategies were assessed: a single round of bead cleanup with a bead/sample ratio of either (i) 0.4 or (ii) 0.8, or (iii) two rounds of cleanup with a bead/sample ratio of 0.4. All cleanup methods significantly improved the A260/230 (ranging from 1.47 to 1.73) and A260/280 (~ 1.90) ratios compared to no additional cleaning (p < 0.05, supplementary Table S1). However, the A260/230 remained substantially lower than 2, so the DNA extracts were considered impure^35^. Additionally, AMPure cleanup resulted in significant loss of DNA in all cases (Table 1 and supplementary Table S1). Interestingly, the A260/230 ratio did not significantly increase with two rounds of cleanup (supplementary Table S1), indicating that method BD might insufficiently remove impurities that also bind to the AMPure XP beads. To address this, we developed method BQ by keeping the battery-powered Omnilyse X bead-beating (B) step but replacing the DNAexpress column purification process with the zymo Quick-DNA HMW magbead (Q) kit (Fig. 1), and found that this increased the A260/230 ratio to 1.91, and improved DNA yield by approximately 500 ng (Table 1). Finally, we assessed if reducing battery voltage in the bead-beating step could improve DNA integrity. Lowering the voltage from 6 to 1.5 V doubled average fragment lengths, increasing them from around 14 to 28 kbp, although this approximately halved DNA yield (Table 1). The final version of protocol BQ consisted of bead-beating using the Omnilyse X tube with 1.5 V, followed by Q purification and one round of AMPure XP bead purification with a 0.4 bead/sample ratio (Fig. 1). Furthermore, it returned comparable amounts of high purity DNA as the current laboratory-based EQ method (enzymatic (E) lysis followed by method Q purification, Fig. 1), although with shorter fragment sizes (28 kbp instead of 58 kbp)^31^.

**Table 1:** Summary of all tested methods and the quality metrics of the resulting DNA extracts.

Method	Lysis method	Bead-beating		DNA purification method	Cleanup and size-selection		A260/280	A260/230	Fragment length (basepairs)	Total extracted DNA (ng)
		Duration (min)	Voltage (V)		Iterations	Bead/Sample ratio				
BD	Bead-beating	3	6	DNAexpress	–	–	1.16 ± 0.35	0.75 ± 0.24	13,728 ± 2863	1592 ± 285
BD	Bead-beating	4	6	DNAexpress	–	–	1.62 ± 0.09	0.88 ± 0.26	12,284 ± 1593	2085 ± 405
BD	Bead-beating	5	6	DNAexpress	–	–	1.46 ± 0.24	0.82 ± 0.29	10,437 ± 1507	2383 ± 1033
BD	Bead-beating	4	6	DNAexpress	1	0.4	1.93 ± 0.02	1.73 ± 0.09	12,826 ± 1377	769 ± 129
BD	Bead-beating	4	6	DNAexpress	1	0.8	1.9 ± 0.08	1.46 ± 0.31	10,877 ± 1167	1062 ± 220
BD	Bead-beating	4	6	DNAexpress	2	0.4	1.91 ± 0.04	1.51 ± 0.09	11,058 ± 565	688 ± 272
BQ	Bead-beating	4	6	QuickDNA	1	0.4	1.91 ± 0.03	2.22 ± 0.08	14,473 ± 1433	1593 ± 165
**BQ**	**Bead-beating**	**4**	**1.5**	**QuickDNA**	**1**	**0.4**	**1.9 ± 0.01**	**2.14 ± 0.12**	**28,311 ± 4252**	**865 ± 257**
EQ	Enzymatic digestion	–	–	QuickDNA	1	0.4	1.77 ± 0.05	1.52 ± 0.19	57,652 ± 2682	1089 ± 172

A260/280 and A260/230 represent the ratio of absorbance at 260 nm divided by absorbance at 280 nm or 230 nm, respectively. The selected optimized on-site applicable protocol is highlighted in bold.

### Performance assessment of different metagenomic workflows

#### Spike-in defined mock community to compare sequencing workflows

To assess the performance of the various metagenomic DNA extraction protocols, aliquots of a single pooled chicken fecal sample were spiked with a DMC consisting of the ZymoBIOMICS Gut Microbiome Standard (GMS), combined with the ZymoBIOMICS Spike-In Control I, which contains two species of marine origin that were not suspected to be present in the fecal background (Table 2)^36,37^. Additionally, we report the ARG content in the DMC reference genomes as detected by ResFinder, and the lateral coverage (the proportion of the reference genome covered by sequencing reads) of each genome over time generated with the final on-site mNGS workflow, in a DMC-spiked chicken fecal sample (Table 2).

**Table 2:** Composition and characteristics of the defined mock community (DMC), along with lateral genome coverage throughout the sequencing run of the DMC spiked into a fecal sample and prepared with on-site method.

Species	Genomic DNA (%)	Genome size (Mb)	GC (%)	Gram Stain	ARG	Lateral coverage over sequencing time of on-site BQV workflow (%)		
						1 h	12 h	24 h
*Faecalibacterium prausnitzii*	13.55	2.91	57.8	+	*tet(W)*	99.5	100.0	100.0
*Veillonella rogosae*	13.58	2.16	39.0	−		100.0	100.0	100.0
*Roseburia hominis*	13.55	3.46	48.7	+ /−		97.4	100.0	100.0
*Bacteroides fragilis*	13.58	5.17	43.3	−	*tet(Q), cep(A)*	99.9	100.0	100.0
Escherichia coli*	13.56	4.95	50.8	−	*mdf(A)*	98.6	100.0	100.0
*Prevotella corporis*	5.82	2.95	44.4	−	*tet(Q)*	99.6	100.0	100.0
*Bifidobacterium adolescentis*	5.82	2.09	59.2	−		70.7	100.0	100.0
*Fusobacterium nucleatum*	5.82	2.45	27.0	−		98.8	100.0	100.0
*Lactobacillus fermentum*	5.83	1.91	52.3	+		45.4	99.7	100.0
*Clostridioides difficile*	1.45	4.21	28.8	+	*erm(B)*	61.0	100.0	100.0
*Akkermansia muciniphila*	1.45	2.85	55.5	−		72.3	100.0	100.0
*Methanobrevibacter smithii*	0.10	1.85	31.0	+		9.6	51.0	73.7
*Salmonella enterica*	0.01	4.76	52.2	−	*aac(6’)-Iaa*	n.d	n.d	n.d
*Enterococcus faecalis*	0.001	2.85	37.5	+	*lsa(A)*	2.6	5.7	7.8
*Clostridium perfringens*	0.0001	3.44	28.3	+		n.d	1.7	2.3
Candida albicans**	0.74	14.68	33.6	n/a		48.7	86.3	88.8
Saccharomyces cerevisiae**	0.67	13.30	38.3	n/a		6.2	51.4	65.3
*Imtechella halotolerans*^+^	2.36	3.00	35.6	−		69.6	100.0	100.0
*Allobacillus halotolerans*^+^	2.12	2.70	39.7	+		41.4	99.6	100.0

*BQV bead-beating–quick-DNA HMW magbead–voltrax sequencing*, *GC* GC-content of the genome, *ARG* antimicrobial resistance gene detected in the reference genome as described in the methods, *n/a* not applicable, *n.d.* not detected.

*E. coli includes 5 strains, lateral genome coverage calculated as mean of template coverages, weighted by mapped base pairs.

**C. albicans and *S. cerevisiae* lateral coverage represent mean coverage of chromosomes weighted by mapped base pairs.

^+^Species from Zymo Spike-in Control I.

#### Protocol impact on sequencing throughput and read length

**Figure 2:**
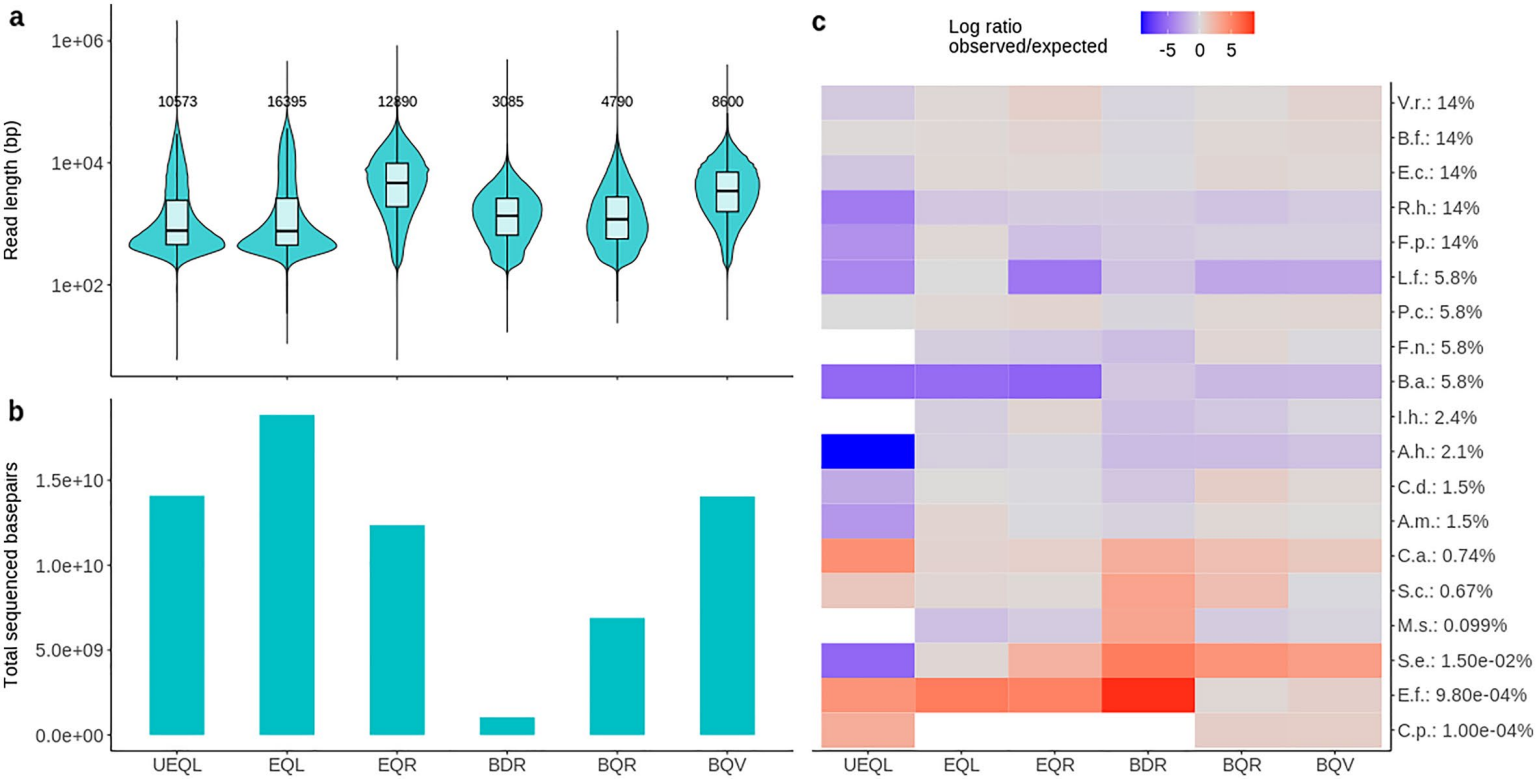
Comparison of sequencing statistics and DMC detection per workflow. (**a**) Read length distributions for the six different sequencing workflows. Numbers above the violin plots indicate the read N50. (**b**) Total sequenced bases by each method. (**c**) Heatmap indicating the ratio of observed versus expected abundance in terms of DNA abundance as stated by the supplier, calculated as described in methods. White tiles indicate that the species was not detected. V.r.: *Veillonella rogosae*, B.f.: *Bacteroides fragilis*, E.c: *Escherichia coli*, R.h.: *Roseburia hominis*, F.p.: *Faecalibacterium prausnitzii*, L.f.: *Lactobacillus fermentum*, P.c.: *Prevotella corporis*. F.n.: *Fusobacterium nucleatum*, B.a.: *Bifidobacterium adolescentis*, I.h.: *Imtechella halotolerans*, A.h.: *Allobacillus halotolerans*. C.d.: *Clostridioides difficile*. A.m.: *Akkermansia municiphila*, C.a.: *Candida albicans*. S.c.: *Saccharomyces cerevisiae*. M.s.: *Methanobrevibacter smithii*, S.e.: *Salmonella enterica*, E.f.: *Enterococcus faecalis*, C.p.: *Clostridium perfringens*. Percentages indicate the theoretical abundance.

To compare the performance of the on-site DNA extraction protocol to other workflows, we generated six nanopore sequencing libraries from the same fecal sample (Fig. 1, Table 3). Briefly, UEQL and EQL were generated with the laboratory-based DNA extraction method EQ combined with ligation sequencing (L), using an unspiked (U) or spiked fecal sample, respectively. EQR was also generated with method EQ, but with transposase-based rapid sequencing (R) instead of ligation sequencing. Library BDR was generated using on-site extraction method BD followed by the R sequencing kit. Next, BQR was generated with the optimal on-site DNA extraction method BQ performed on the Bento Bio Pro device, with a 1.5 V battery replacing the 6 V battery in BD. Finally, the complete, optimized on-site sequencing workflow (BQV) consisted of 1.5 V bead-beating lysis (B), Q purification performed on the Bento Bio Pro, followed by automated library preparation on the VolTRAX device, using the complete DNA extract in the transposase-based VolTRAX sequencing kit (V). In all cases, additional purification using a ratio of 0.4 volumes of AMPure XP beads to 1 volume of DNA extract was performed on the DNA extracts before continuing with library preparation. The aggregate sequencing output and statistics of the various protocols were compared (Table 3, Fig. 2). In general, the enzymatic lysis libraries (UEQL, EQL and EQR) outperformed the bead-beating libraries (BDR, BQR, BQV) in terms of sequencing throughput (Fig. 2b), with exception of BQV. They also demonstrated higher read length N50 (Fig. 2a). However, bead-beating methods were 1–2.5 h faster to execute (Table 3, Fig. 1). Between the enzymatic lysis libraries, EQL showed a broad distribution of read lengths, with low median but high N50, while EQR is characterized by higher median, but lower N50 (Fig. 2a). Interestingly, a rapid decrease in available pores was observed for on-site method BDR following flow cell loading (supplementary Fig. S1). This rapid drop-off in number of available pores did not occur for the other on-site methods (BQR and BQV), where the obtained DNA was of higher purity, increasing sequencing throughput. The reduced bead-beating intensity in BQR and BQV improved DNA fragment sizes, resulting in higher read lengths and throughput compared to BDR. Finally, the higher amount of input DNA in BQV, along with the additional automated magnetic bead cleanup in the VolTRAX library preparation, further increased read lengths and throughput, resulting in comparable throughput to the laboratory-based library EQR, although read N50 remained lower.

**Table 3:** Overview of sequencing workflows.

Workflow	Sample	Microbial lysis method	DNA purification	A260 /280	A260 /230	Fragment length (bp)	DNA input (ng)	Nanopore library preparation	Time required (h)	Total bases (Gb)	Read N50
UEQL	Chicken feces	E	Q	1.83	1.55	60,000	820.8	SQK-LSK109	4.5–5	14.1	10,573
EQL	Chicken feces spiked with DMC	E	Q	1.83	2.03	50,500	999.4	SQK-LSK109	4.5–5	18.9	16,395
EQR							380.0	SQK-RAD004	4–4.5	12.4	12,890
BDR		B (4 min, 6 V)	D	1.81	1.28	9852	382.5	SQK-RAD004	2–2.5	1.1	3085
BQR		B (4 min, 1.5 V)	Q, on Bento Bio	1.88	2.32	27,288	296.25	SQK-RAD004	2.5–3	6.9	4790
BQV				1.87	1.87	38,101	1097.3	VSK-VSK004	2.5–3	14.0	8600

*UEQL* Unspiked–enzymatic lysis–quick-DNA magbead HMW-ligation sequencing, *EQL* enzymatic lysis–quick-DNA magbead HMW–ligation sequencing, *EQR* enzymatic lysis–quick-DNA magbead HMW—rapid sequencing, *BDR* bead-beating–DNAexpress–rapid sequencing, *BQR* bead-beating–quick-DNA HMW magbead–rapid sequencing, *BQV* bead-beating–quick-DNA HMW magbead–voltrax sequencing.

#### Sequencing workflow impacts taxonomic classification

As we used a single pooled chicken fecal sample for all methods, spiked with the same DMC, this allowed us to identify any possible taxonomic bias introduced by the experimental workflow. We mapped the reads generated with each method to a database with the DMC reference genomes, and compared the observed relative abundance (RA) for each DMC species to its theoretical relative abundance (TRA) in the spike-in DMC (supplementary Table S2, Fig. 2). In the unspiked sample UEQL, containing no DMC, the species *Bacteroides fragilis, Prevotella corporis, Candida albicans, Enterococcus faecalis* and *Clostridium perfringens* were detected in high or similar amounts compared to the DMC, while most other DMC species were absent or observed in low abundance. *Imtechella halotolerans* and *Allobacillus halotolerans* were not observed, or observed at a very low level, respectively (Table S2). For the fecal sample spiked with DMC, *Roseburia hominis* and *Bifidobacterium adolescentis* were systematically underrepresented, but the bias was most pronounced for *Bifidobacterium adolescentis* in EQL and EQR, where the observed RAs were more than 100-fold diminished compared to the expected values (supplementary Table S2). For *Lactobacillus fermentum*, the RA was mainly diminished in EQR, but also in BDR, BQR and BQV. Method BDR showed reduced RA for DMC species down to 1.5% of TRA, while the species with TRA < 1.5% were detected more than expected. Further investigation of the mapped reads showed effects of the sequencing workflows on the read length distribution per species (supplementary Fig. S2). Briefly, enzymatic methods generated longer reads for almost all species, with exception of *L. fermentum*, and showed large variations in read length distributions between species. In contrast, bead-beating methods generally resulted in smaller read lengths, with length distributions between species showing less variation. Next, we additionally used a broad taxonomic database to further investigate the detected fecal microbiome background for all workflows. Compared to the unspiked background (UEQL), several background species such as *Bifidobacterium gallinarum* and members of the *Lactobacillaceae* family are underrepresented in enzymatic lysis workflows (EQL, EQR). For the bead-beating methods (BDR, BQR, BQV), this is not the case (supplementary Fig. S3).

### The on-site workflow enables rapid taxonomic identification

**Figure 3:**
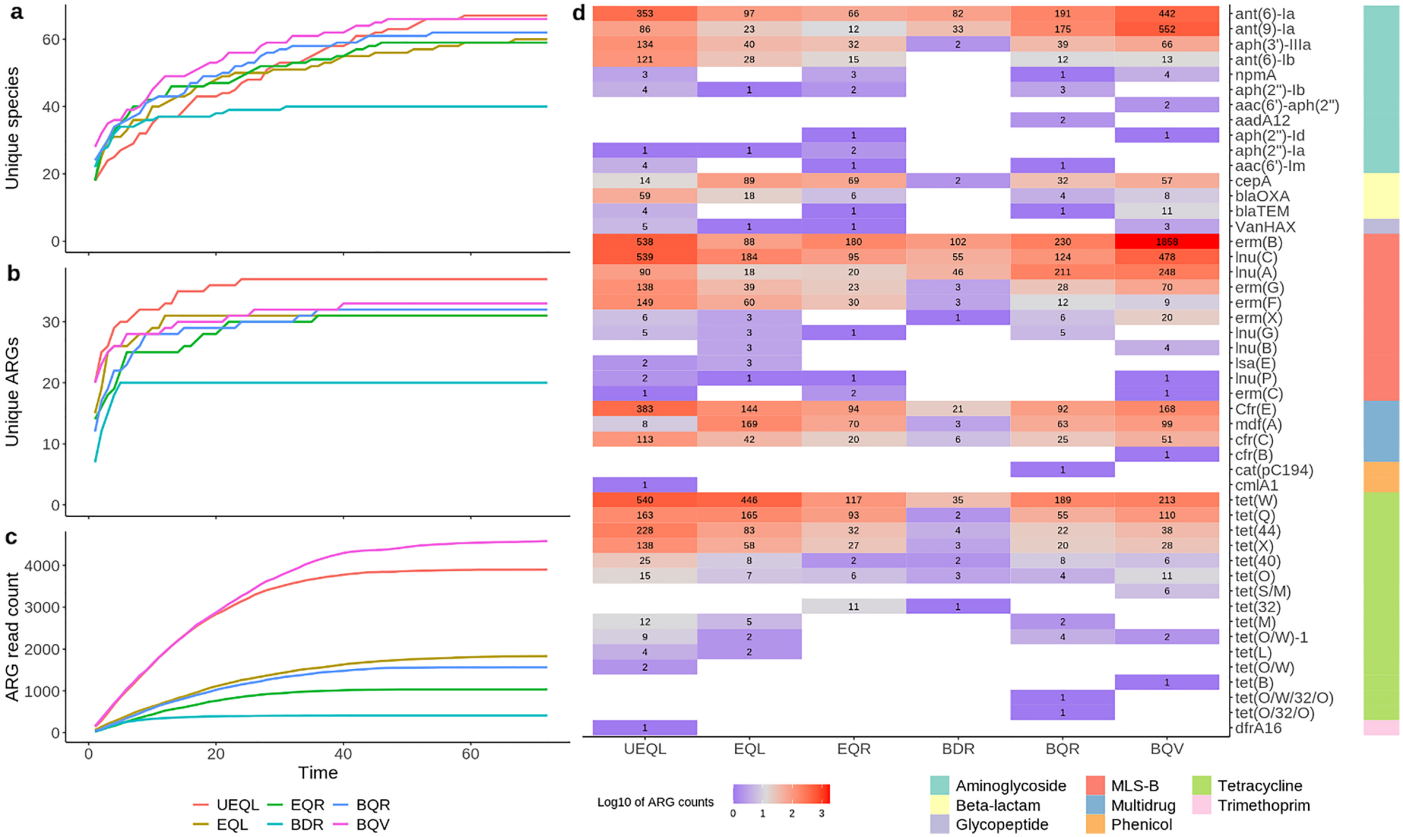
Comparison of general species and ARG detection between workflows (**a**) Number of unique species detected with 5% template coverage and 80% query identity throughout the sequencing run. (**b**) Number of unique resistance genes (ARG) detected in full with 97% template identity. (**c**) Number of reads containing full-length ARGs, with 97% template identity cutoff. (**d**) Full length ARG counts by workflow, ARG and class of antimicrobial resistance (legend in color bar). White tiles indicate that the ARG in question was not detected.

Given a spiked-in taxon with known genome size and abundance, and the time at which each mapped read was sequenced, the sequencing run time required to reach a certain genome coverage can be determined. As such, the lateral coverage for each taxon in the DMC spiked into a fecal background at 1, 12, and 24 h was determined for the best performing on-site workflow, BQV (Table 2). Most DMC species with RA > 5% reached near-complete genome coverage within the first hour of sequencing, with exception of *B. adolescentis* and *L. fermentum*, which are both underrepresented in the overall sequencing data (Fig. 2). However, these species had been fully sequenced after 12 h, along with species with abundances between 1 and 5%. For the yeasts *C. albicans* and *S. cerevisiae*, 100% genome coverage is never reached. Similarly, the coverage for *M. smithii* did not reach saturation. Finally, *S. enterica* was not detected, while *E. faecalis* and *C. perfringens*, having RAs > 1000-times that of *S. enterica* in fecal background UEQL (Table S2), did not reach coverages above 10%. Next, by using a broad taxonomic database, on-site method BQV was found to outperform the other spiked sample runs in terms of number of uniquely identified species (Fig. 3a). Verifying whether this concerned true positive species was impossible due to the unknown fecal background composition. Ultimately, all methods converged to a number of ca. 60 species, except for method BDR which stabilized earlier at a lower level.

### Resistome profiling using full-length resistance genes in single reads

To profile the ARG content, we sought to identify reads containing full-length ARGs by mapping them to the ResFinder database, with stringent filtering of 97% identity to the complete ARG template. The optimal on-site method, BQV, quickly picked up many types of ARGs, ultimately detecting a similar or higher number of unique ARGs as most other methods. The highest ARG diversity was found in the unspiked background UEQL (Fig. 3b). Next, we compared the resistance profiles between the different workflows. Among workflows performed on the spiked fecal sample, BQV had the highest ARG read count, followed by methods EQL, BQR, EQR and BDR (Fig. 3c). The enzymatic methods returned high read count proportions of ARGs present in the DMC, and generally lower proportions of ARG classes exclusive to the fecal background (UEQL) (supplementary Fig. S4). In contrast, the on-site workflows using bead-beating returned a combination of background and DMC ARG classes, with exception of method BDR, which returned a low overall number of ARG reads (Fig. 3d, supplementary Fig. S4). In summary, most ARGs detected in the fecal background (UEQL) are consistently detected by other workflows, albeit at different abundances. Compared to the other workflows, a substantially larger part of the ARG reads generated with method BQV contained the *erm(B)* gene, although it was detected in high RA in all cases.

### Genomic context links resistance genes to their hosts

**Figure 4:**
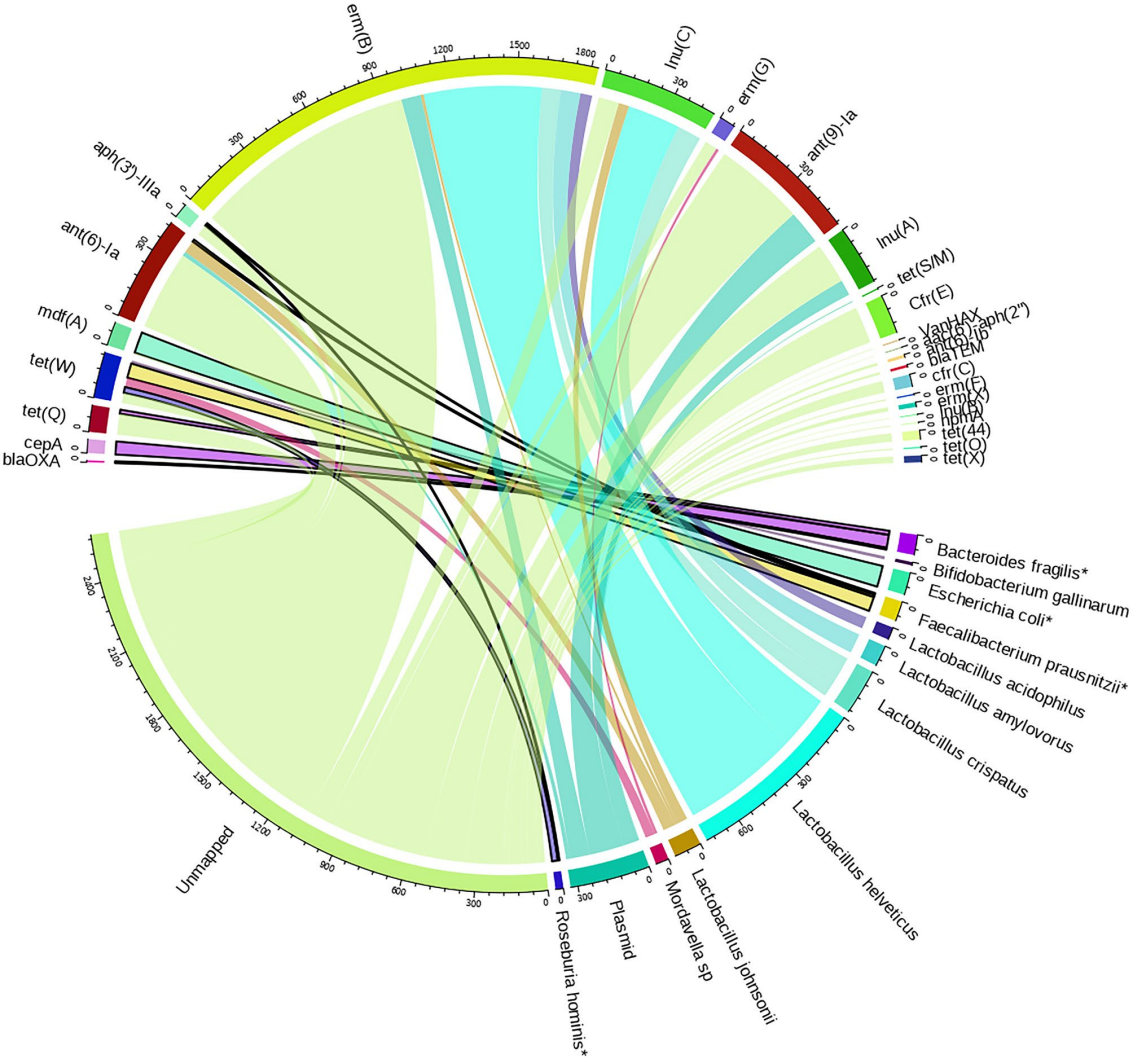
Resistance gene–host links generated with on-site sequencing method BQV. Read counts are indicated by the circular scale. Connections from ARG to spike-in DMC members are highlighted in bold. *: Members of the spiked-in DMC.

As long reads can provide additional information on the flanking regions of detected ARGs, we attempted to link ARGs to their microbial host species. To do so, we retrieved reads carrying full-length ARGs from their alignment to the broad taxonomic database. Optimal on-site method BQV generated the most of such combinations (Fig. 4, supplementary Figs. S4–S9). Across all experiments, more than half of the full-length ARG reads could not be linked to any host. According to the UEQL background, most of the identified ARG-hosts native to the fecal sample belong to the *Lactobacillaceae* family, with links to aminoglycoside and MLS-B resistance genes, followed by members of the *Mordavella* and *Bacteroides* genera with tetracycline resistance, including *Bacteroides fragilis* (a DMC member) which also contains beta-lactam and aminoglycoside ARGs. For the spiked samples, the enzymatic lysis workflows EQL and EQR mainly identified ARG-host links from the DMC members (supplementary Figs. S5 and S6) with high RA, with limited connections between ARGs and background species. Specifically, the aminoglycoside and MLS-B ARG carrying *Lactobacillaceae* from the background were strongly diminished. In contrast, the bead-beating workflows BQR and BQV demonstrated a higher diversity of ARG-host links (supplementary Figs. S8 and S9), primarily detecting background ARG-host combinations as well as several ARG-host combinations from the DMC. Additionally, these methods resulted in a higher number of ARGs being placed onto plasmids compared to the background. The DMC members *P. corporis* (absent from database)*, S. enterica* and *E. faecalis* (< 0.01% TRA) were not detected as ARG hosts by any of the workflows. Interestingly, the DMC member *R. hominis* is reported as containing the ARGs *aph(3’)-IIIIa* and *tet(W)* in several of the datasets, while these genes were not detected in the reference genome of the DMC strain as supplied by the manufacturer. However, these ARGs were reported in the *R. hominis* genome present in the larger database (accession NZ_LR699011.1, supplementary Table S3).

## Discussion

The development of real-time, high-throughput sequencing on portable devices has opened up the possibility of rapid, comprehensive and culture-free diagnostics and surveillance using mNGS. However, the DNA extraction and purification protocols preceding mNGS often still require centralized laboratories and high initial investments. Additionally, sample processing, DNA extraction and library preparation have been shown to affect the reliability of the results^27,38^. In this study, we have developed an on-site applicable DNA extraction and library preparation method for nanopore sequencing that is comparable or superior to a current laboratory-based protocol^31,39^. We also demonstrated the use of a spike-in defined mock community to compare workflows, and found large variations in taxonomic composition and ARG content, highlighting the importance of using appropriate DNA extraction and sequencing methods. 

The optimized on-site applicable method (BQV) consists of a portable bead-beating cell lysis approach, combined with portable magnetic bead-based DNA purification. With the inclusion of an additional cleanup and size-selection step, the method consistently delivers high yields of high purity, high molecular weight DNA. In combination with the laptop-powered, portable VolTRAX device, high quality nanopore libraries can be obtained. As such, the final protocol allows for on-site mNGS library generation from complex samples in 3 h or less, by using portable devices for both cell lysis, DNA purification and library preparation (Fig. 1). Furthermore, the resulting library generated a similar sequencing throughput as current laboratory methods based on enzymatic lysis (EQL, EQR), while bypassing time-consuming incubation steps (Fig. 1)^31,39^. Initially, the Claremont Bio DNAexpress method (BD, Fig. 1) was considered for DNA extraction because of its rapidness, portability, and prior results showing high DNA yields^31^. However, we observed it returned DNA libraries of poor quality, which was resolved by replacing the DNAexpress purification step with the Quick-DNA HMW MagBead kit. This modification improved purity, thereby reducing flow cell degradation, highlighting the importance of sufficient contaminant removal for mNGS applications^33,40^. Additionally, reducing bead-beating voltage increased DNA fragment sizes, but lowered yield, indicating a trade-off between DNA yield and fragment lengths depending on bead-beating intensity. Similarly, enzymatic lysis methods generated higher read lengths than bead-beating methods, probably due to the lower mechanical stresses. We also assessed differences between the adapter ligation (L), and transposase adapter (R) library preparation kits. The differences we found are similar to previous findings by Tvedte et al.^41^. However, another study showed no clear effect of library preparation method on resulting read lengths^42^. 

Overall, the choice of mNGS workflow substantially impacted the resulting resistome and taxonomic profiles, with our optimal on-site method (BQV) detecting most of the spiked DMC, along with high ARG and taxonomic diversity from the background sample. However, several of the spike-in DMC members were systematically over- or underrepresented, independent of the method used. On one hand, the presence of sample (background) strains that are closely related to spike-in strains likely results in their overrepresentation, as exemplified by some of our spike-in species which can be found in the chicken gut microbiome^38,43,44^. This issue can be avoided by using spike-in species foreign to the sample, which we demonstrated by using *A. halotolerans* and *I. halotolerans*. In other cases, the use of a limited database with only spike-in genomes could have resulted in false positive mapping of fecal background reads to the DMC genomes, increasing their apparent abundance. Such errors could be a result of the applied read mapping approach, which only returns a single, best mapping template sequence, while a lowest common ancestor approach would be more accurate in ambiguous cases. On the other hand, underrepresentation of spike-in strains can occur either because they are present below the detection limit, or because of challenges in obtaining DNA of sufficient quality and quantity from those strains. As an example of the latter, *B. adolescentis* was severely depleted when using enzymatic lysis, which has also been observed for *Bifidobacterium* in previous studies^4,39,45^. Additionally, *L. fermentum* was underrepresented in experiments using transposase-based sequencing methods (R or V), while showing short read lengths upon enzymatic lysis, as previously also found by Martin et al^39^. The combination of enzymatic lysis with transposase library preparation (EQR) most severely diminished observed *L. fermentum* abundance, likely due to the aggravating effects of both methods on read lengths^46^. These method-specific biases extended to the fecal background, where *Lactobacillaceae* family members and *Bifidobacterium gallinarum* were severely underrepresented after enzymatic lysis. In general, bead-beating methods such as in the final optimal on-site method BQV produced less severe biases and more uniform read lengths across organisms, at the cost of achievable read lengths. These findings indicate trade-offs between lysis efficiency, accurate taxonomic representation, and DNA integrity, likely due to varying cell wall degradation resistance across species^38,47^. Similar method-specific batch effects have been demonstrated before, and could explain large discrepancies in taxonomic profiles between studies using different DNA extraction methods^38,43,48,49^. Importantly, *Lactobacillaceae* have been reported to be among the dominant members of the chicken microbiome, and the inability to lyse and sequence them could therefore be problematic^38,43,49–51^. Here, we additionally found method-specific effects on resistome profiles. Using the sequence information provided by long reads on ARG flanking regions, ARG host species could be inferred, allowing to trace back resistome differences to the method-specific taxonomic differences mentioned before. In the case of the chicken fecal samples analyzed here, variation in *Lactobacillaceae* detection across methods affected ARG profiles. However, a large part of the ARGs could not be attributed to any host organism. This could reflect limits in database contents, such as the absence of certain microbial strains or mobile elements, which are known to frequently carry ARGs^52–54^. Additionally, it needs to be mentioned that ARG-host connections should be interpreted with caution. First, as ARGs can be highly identical to each other or to wild type genes, we used a 97% identity threshold to the full-length ARG template to reduce misclassification^54^. Second, sequencing errors can reduce both ARG and taxonomic classification performance. Improved nanopore sequencing accuracy (i.e. with the R10.4.1 flow cell) could improve both ARG and taxonomic classification performance^55–57^. Additionally, (integrative) mobile elements and the choice of database can confound the analysis, as was demonstrated for *R. hominis* in our spike-in DMC^52,53^. Furthermore, ARGs are frequently located in mobile genetic elements, which are often absent from taxonomic databases as they are difficult to attribute to any particular host. To enhance the reliability of ARG-host attribution, future developments should therefore focus on refining taxonomic databases and improving classification algorithms. Adapting these computational methods for applicability in low resource settings might be needed to perform a complete sample to result workflow in situ. 

In summary, we developed and optimized a rapid DNA-extraction and sequencing workflow (BQV), applicable for on-site nanopore mNGS in the context of pathogen diagnostics and surveillance, yielding DNA libraries of comparable quality to current laboratory-based protocols. Using a defined mock community spiked into fecal samples, we further assessed the quality of this method in terms of taxonomic and resistome profiling. By demonstrating rapid detection of both spike-in species and background diversity, we illustrate how detection times could be further reduced. Although we focused on chicken fecal samples here, we believe our method could also be applied to other complex sample types. More dilute samples could require an additional concentrating step, or the use of PCR-based library preparation. Overall, bead-beating was found to be the fastest lysis approach, while also giving a comprehensive view on sample taxonomic and resistome content. However, bead-beating intensity and duration should be optimized depending on the sample type being investigated, to find appropriate parameters that ensure effective cell lysis and DNA extraction, while maintaining long fragment sizes. If short-read mNGS would be used, the latter is less critical, although this would not provide results on-site and in real-time^33,38^. Additionally, spike-in controls can allow for direct comparison across methods, but should contain microbial species foreign to the sample, and with varying cell wall composition. Finally, we demonstrate the added value of long-read metagenomic sequencing in identifying full-length ARGs and leveraging the additional sequence information to attribute them to a host. This paper focused on a workflow to generate real-time sequencing data by nanopore sequencing, applicable on-site. To analyze the data, a stable internet connectivity or connection to the electrical grid were still used. To obtain a full on-site applicable mNGS workflow covering data generation and analysis, complementary studies could focus on developing real-time computational pipelines, including adapted/downscaled databases, that can be performed on laptops or other portable devices. 

## Methods

### Sample collection and spiking

Chicken fecal samples were collected and processed as follows: one spoonful of fecal material (≈ 1 g) was collected and stored in a DNA/RNA Shield™ Fecal Collection Tube R1101 containing 9 ml of DNA/RNA-shield (Zymo Research, Irvine, CA, USA), according to the manufacturer’s instructions. The sample was mixed well by vortexing extensively, and distributed in 1 ml aliquots. These were then centrifuged for 2 min at 5000*g*, after which the supernatant was stored separately. For comparison of metagenomic workflows, aliquots of 100 mg each were made from a single pooled fecal sample, and were recombined with 100 µL of the pooled supernatant from the previous step. Several of the aliquots were spiked with the spike-in DMC, consisting of 75 µl of ZymoBIOMICS Gut Microbiome Standard (D6331), along with 7.8 µl Zymo Spike-in control I (D6320) (Zymo Research, Irvine, CA, USA) (Table 2).

### Development of on-site DNA extraction protocol

The Claremont Biosolutions DNAexpress method (method BD) was performed according to manufacturer’s instructions with the following adaptations: to the Omnilyse X™ bead-beating tube (Claremont Biosolutions, Upland, CA, USA), 2X Buffer Reagent 1 (Claremont Biosolutions, Upland, CA, USA), 20 µL of Proteinase K (20 mg/mL), 2.5 µL of 1 M DTT was added along with the sample. Bead-beating was performed for varying durations using the provided 6 V or 1.5 V batteries (Table 1). During the bead-beating, the tube was inverted several times for better homogenization. After lysis, the Proteinase K digestion was incubated for 30 min at room temperature. DNA was then purified with either the DNAexpress column according to manufacturer’s instructions (method BD), or with the Quick-DNA HMW magbead kit (Zymo Research, Irvine, CA, USA), with several modifications (method BQ): the lysed and proteinase-K digested sample was centrifuged at 6000*g* for 2 min, and the supernatant was transferred to a 1.5 mL tube. Thirty-three µL of MagBeads and 800 µL of Magbinding buffer (Zymo Research, Irvine, CA, USA) were added, after which the sample was put on a Hula mixer for 20 min. After the final washing step, the beads were dried at 55 °C for 7 min. Finally, the DNA was eluted at 55 °C for 10 min in a dry-bath. For some experiments, additional cleanup of the eluted DNA was performed using Agencourt AMPure XP beads (Beckman Coulter, Indianapolis, IN, USA) according to the following protocol: beads were added to the samples in varying ratios (Table 1), followed by 5 min incubation on a Hula mixer. The beads where then washed twice with freshly prepared 80% ethanol (Merck Millipore, Darmstadt, Germany). Finally, the beads were resuspended in 15 µL nuclease-free water (Thermo Fisher Scientific, Waltham, MA, USA), and incubated for 5 min at 55 °C to elute the DNA.

### Statistical tests to compare DNA extraction protocols

Statistical comparisons of the DNA extraction protocols were performed in R (v4.2.2)^58^, using the rstatix package (v0.7.1). Normality and equality of variance assumptions were tested using the Shapiro–Wilk and Bartlett’s tests, respectively. When these assumptions were met, ANOVA was used, followed by Tukey’s HSD test upon significance. In other cases, the Kruskal–Wallis test was used, followed by Dunn’s post-hoc test with Benjamini–Hochberg correction for multiple testing after a significant Kruskal–Wallis test.

### Laboratory-based protocol

The laboratory-based extraction method consisted of enzymatic lysis and Quick-DNA HMW Magbead purification (EQ)^31^. Enzymatic lysis was performed according to the microbial lysis method in the Quick-DNA HMW MagBead Kit protocol (Zymo Research, Irvine, CA, USA) with the following modifications: Cell wall digestion was performed using 100 µL of Tris–HCl buffer and 20 µL of Metapolyzyme lytic enzyme mixture (Sigma-Aldrich, Saint Louis, MO, USA), with incubation at 37 °C for 1 h. After the lysis step, 20 µL of 10% SDS and 10 µL of Proteinase K were added, followed by incubation at 55 °C for 30 min. Magnetic bead purification was done with the Quick-DNA HMW magbead kit as described for method BQ. Further cleanup was done with Agencourt AMPure XP beads (Beckman Coulter, Indianapolis, IN, USA), carried out as described above, with bead to sample ratio of 0.4.

### DNA quality and quantity

DNA purity, quantity, integrity and fragment lengths were measured as follows: purity was assessed by measuring the A260/280 and A260/230 ratios with the Nanodrop® 2000 (Thermo Fisher Scientific, Waltham, MA, USA) spectrophotometer. The quantity was measured using the Qubit™ dsDNA BR Assay kit on a Qubit™ 4 Fluorometer (Invitrogen by Thermo Fisher Scientific, Waltham, MA, USA). DNA integrity and fragment lengths were determined by capillary gel electrophoresis on the Tapestation 4200, using the genomic DNA screentapes and reagents (Agilent Technologies, Palo Alto, CA).

### Nanopore sequencing

Various libraries were made from the spiked feces (Table 3). First, the laboratory-based DNA extraction protocol EQ was performed as described above, using a 0.4 ratio of AMPure XP beads (Beckman Coulter, Indianapolis, IN, USA) to sample in the final purification step. The obtained DNA extract was used in combination with the ONT ligation sequencing kit (SQK-LSK109, Oxford Nanopore Technologies, Oxford, UK) to make the EQL sequencing library, or with the rapid sequencing kit (SQK-RAD004, Oxford Nanopore Technologies, Oxford, UK) to generate the EQR library. Another library, UEQL, was generated from an unspiked fecal aliquot extracted with method EQ, purified using a 0.4 bead to sample ratio of AMPure XP beads (Beckman Coulter, Indianapolis, IN, USA) and was further prepared using the ligation sequencing kit (SQK-LSK109, Oxford Nanopore Technologies, Oxford, UK). Regarding the on-site DNA extraction methods, method BD was purified using a 0.4 bead to sample ratio of AMPure XP beads (Beckman Coulter, Indianapolis, IN, USA), followed by the rapid sequencing kit (SQK-RAD004, Oxford Nanopore Technologies, Oxford, UK) to generate library BDR. Method BQ was used to generate libraries BQR and BQV, by purifying the extracts using a 0.4 bead to sample ratio of AMPure XP beads (Beckman Coulter, Indianapolis, IN, USA), followed by either the rapid (SQK-RAD004) or VolTRAX (VSK-VSK004, Oxford Nanopore Technologies, Oxford, UK) sequencing kits, respectively. Additionally, bead-beating in method BQ was performed using the Omnilyse X tube (Claremont Biosolutions, Upland, CA, USA) powered by a 1.5 V battery, instead of the 6 V battery used for BD. All sequencing kits were used according to manufacturer’s instructions, with an amount of input DNA corresponding to kit requirements or the maximum amount of available DNA. In case of the Voltrax sequencing kit—all extracted DNA was used. During the ligation sequencing kit (SQK-LSK109, Oxford Nanopore Technologies, Oxford, UK), the Short Fragment Buffer was used during the adapter ligation and cleanup step. All heating and centrifugation steps in BQ were performed on the Bento Bio Pro (Bento Bioworks, London, UK), which is a portable device that includes a thermocycler and microcentrifuge. The BQV library was prepared automatically by the VolTRAX V2b device (Oxford Nanopore Technologies, Oxford, UK) by loading all obtained DNA along with the required VolTRAX sequencing kit reagents (VSK-VSK004, Oxford Nanopore Technologies, Oxford, UK), after which the device automatically carries out the required mixing, thermocycling and magnetic bead cleanup. Finally, all libraries were sequenced on a single R9.4.1 (FLO-MIN106, Oxford Nanopore Technologies, Oxford, UK) flow cell per library, for 72 h on a GridION Mk1 device (Oxford Nanopore Technologies, Oxford, UK).

### Nanopore basecalling and QC

Guppy v5.0.7 was used for basecalling raw nanopore data, using the super accuracy (sup) basecalling model dna_r9.4.1_450bps_sup.cfg. The custom script GetFastqStats.py was then used to summarize read statistics per experiment. For further processing, reads were filtered using Nanofilt v2.8^59^, removing reads with Q-score < 7 and length < 300.

### Taxonomic analysis

KMA v1.4.4 was used to construct an indexed database from the DMC with the GMS and Spike-in control I reference genomes (provided by the manufacturer: https://zymoresearch.eu/collections/zymobiomics-microbial-community-standards) using the kma index command^60^. Another broad in-house database was made with the same command, and contained all NCBI RefSeq genome entries with the “complete genome” assembly level (database accessed February 11, 2021^61^), and accession prefixes NC, NW, AC, and NZ of the following taxonomic groups: archaea, bacteria, fungi, protozoa, and viruses. Filtered reads were then mapped to these databases with the following options: -mem_mode -bc 0.7 -bcNano -ID 0.0 -ef -proxi 0.9 -na -nc -nf -1t1 -ca -sam. The res and mapstat summary files produced by KMA were aggregated and processed with the KMA_taxa_summary.py python script (v3.10.5) for taxonomic alignments. This script uses the Template_parsing.py script to parse template sequence names and groups the results on the species level. Template and query coverages and identity were aggregated by taking the means of these statistics across the different species templates, weighted by the bases mapped to those templates. Template sequences labelled as phage, virus or plasmid were not grouped together with their host species. Observed relative abundances for the DMC species were then calculated by dividing the amount of mapped bases to one species by the total mapped bases to the complete DMC database. The sam output generated by KMA were sorted and converted to bam format using samtools v1.9, and further processed with custom python scripts using the pysam module (v0.19.1): GetCoverageByTime.py, to summarize classification statistics over time and Readlevel_align_stats.py to calculate species-level sequencing statistics.

### Resistome profiling

To identify the ARG composition of the DMC, the spike-in DMC reference genomes were mapped to the ResFinder database with KMA, using the same options as for taxonomic mapping, excluding the -1t1 option^60,62^. Reads from the various workflows were mapped with the same method. The custom python script GetAMRlinksByTime.py was used to retrieve reads containing ARGs, requiring 97% identity to the full-length ResFinder database template. Additionally, this script retrieved the same reads from the alignment against the taxonomic databases, generating tabular outputs summarized by combination of ARG and taxonomic templates. To consider a reported ARG-host combination as likely true, a total of 50 kbp of matching bases between ARG reads and a taxonomic template was set as a cutoff value.

### Data visualization

Summarized data were visualized in R (v4.2.2) with the ggplot2, ggpubr and circlize libraries^58,63–65^. The custom script Plot_SeqRunStats.R was used to generate figures on sequencing summary statistics and taxonomic heatmaps, Species_ARG_ByTime.R generated figures on species and ARG detection over time, as well as ARG content. The custom script AMRlinkPlots generated the chord diagrams on ARG-host combinations.

### Supplementary Information

Supplementary Information.

## Data Availability

The sequencing data supporting the conclusions of this article is available in the NCBI Sequence Read Archive (SRA) repository, under the BioProject ID: PRJNA1011201.

## Code availability

The custom scripts used to perform the analyses carried out in this study are available in the repository: https://github.com/brambloemen/FARMED_onsite.
