# Supplementary material for: Development of a portable on-site applicable metagenomic data generation workflow for enhanced pathogen and antimicrobial resistance surveillance
Source: Sci Rep. 2023 Nov 11;13:19656. doi: 10.1038/s41598-023-46771-z (PMC10640560; doi:10.1038/s41598-023-46771-z)
Supplement: Supplementary file 1 — Supplementary Information. [file 41598_2023_46771_MOESM1_ESM.pdf]

Supplementary information for:

Development of a portable on-site applicable metagenomic data generation workflow for enhanced pathogen and antimicrobial resistance surveillance

Bram Bloemen[1,2], Mathieu Gand[1], Kevin Vanneste[1], Kathleen Marchal[2,3], Nancy H.C. Roosens[1], and Sigrid C. J. De Keersmaecker[1,*]

[1] Transversal activities in Applied Genomics, Sciensano, rue Juliette Wytsman 14, 1050 Brussels, Belgium

[2] Department of Information Technology, IDLab, Ghent University, IMEC, 9052 Ghent, Belgium

[3] Department of Plant Biotechnology and Bioinformatics, Ghent University, 9052 Ghent, Belgium

[*] Sigrid.dekeersmaecker@sciensano.be

Variable	Statistical test	p-value	Post-hoc test	Results from post-hoc pairwise test between protocol iterations	p -value
A260/230	ANOVA	4.29e-20	Tukey HSD	BD without cleanup, 3 versus 4 minutes bead-beating at 6V	1.00
				BD without cleanup, 3 versus 5 minutes bead-beating at 6V	1.00
				BD without cleanup, 4 versus 5 minutes bead-beating at 6V	1.00
				**BD 4 minutes bead beating at 6V, no versus 0.4x bead/sample AMPure**	**2.75e-05**
				**BD 4 minutes bead beating, no versus 0.8x bead/sample AMPure**	**3.37e-04**
				**BD 4 minutes bead beating, no versus 2 rounds of 0.4x bead/sample AMPure**	**3.83e-05**
				BD 4 minutes bead beating, 0.8x bead/sample AMPure versus 0.4x bead/sample AMPure	0.82
				BD 4 minutes bead beating, 1 versus 2 rounds of 0.4x bead/sample AMPure	0.94
				BD 4 minutes bead beating, 1 round 0.8x bead/sample AMPure versus 2 rounds of 0.4x bead/sample AMPure	1.00
				BD 4 minutes bead beating at 6V, 1 round 0.4x bead/sample AMPure with DNAexpress purification versus same method with QuickDNA purification	0.26
				BD 4 minutes bead beating at 6V, 1 round 0.4x bead/sample AMPure with QuickDNA purification versus same method using 1.5V bead beating	0.36
				**BQ 4 minutes bead beating at 1.5V, 0.4x bead/sample AMPure compared to EQ with 0.4x bead/sample AMPure**	**2.03e-04**
A260/280	Kruskal-Wallis	9e-10	Dunn's with Benjamini-Hochberg correction	BD without cleanup, 3 versus 4 minutes bead-beating at 6V	0.284
				BD without cleanup, 3 versus 5 minutes bead-beating at 6V	0.61
				BD without cleanup, 4 versus 5 minutes bead-beating at 6V	0.61
				**BD 4 minutes bead beating at 6V, no versus 0.4x bead/sample AMPure**	**9.60e-03**
				**BD 4 minutes bead beating, no versus 0.8x bead/sample AMPure**	**5.90e-03**
				**BD 4 minutes bead beating, no versus 2 rounds of 0.4x bead/sample AMPure**	**4.05e-03**
				BD 4 minutes bead beating, 0.8x bead/sample AMPure versus 0.4x bead/sample AMPure	0.76
				BD 4 minutes bead beating, 1 versus 2 rounds of 0.4x bead/sample AMPure	0.76
				BD 4 minutes bead beating, 1 round 0.8x bead/sample AMPure versus 2 rounds of 0.4x bead/sample AMPure	0.96
				BD 4 minutes bead beating at 6V, 1 round 0.4x bead/sample AMPure with DNAexpress purification versus same method with QuickDNA purification	0.764
				BD 4 minutes bead beating at 6V, 1 round 0.4x bead/sample AMPure with QuickDNA purification versus same method using 1.5V bead beating	0.97
DNA yield	Kruskal-Wallis	9.8e-08	Dunn's with Benjamini-Hochberg correction	BD without cleanup, 3 versus 4 minutes bead-beating at 6V	0.49
				BD without cleanup, 3 versus 5 minutes bead-beating at 6V	0.52
				BD without cleanup, 4 versus 5 minutes bead-beating at 6V	0.93
				**BD 4 minutes bead beating at 6V, no versus 0.4x bead/sample AMPure**	**1.4e-02**
				**BD 4 minutes bead beating, no versus 0.8x bead/sample AMPure**	**2.3e-02**
				**BD 4 minutes bead beating, no versus 2 rounds of 0.4x bead/sample AMPure**	**1.7e-03**
				BD 4 minutes bead beating, 0.8x bead/sample AMPure versus 0.4x bead/sample AMPure	0.53
				BD 4 minutes bead beating, 1 versus 2 rounds of 0.4x bead/sample AMPure	0.98
				BD 4 minutes bead beating, 1 round 0.8x bead/sample AMPure versus 2 rounds of 0.4x bead/sample AMPure	0.35
				BD 4 minutes bead beating at 6V, 1 round 0.4x bead/sample AMPure with DNAexpress purification versus same method with QuickDNA purification	0.14
				BD 4 minutes bead beating at 6V, 1 round 0.4x bead/sample AMPure with QuickDNA purification versus same method using 1.5V bead beating	0.15
Fragment length	Kruskal-Wallis	4.7e-09	Dunn's with Benjamini-Hochberg correction	BD without cleanup, 3 versus 4 minutes bead-beating at 6V	0.74
				BD without cleanup, 3 versus 5 minutes bead-beating at 6V	0.21
				BD without cleanup, 4 versus 5 minutes bead-beating at 6V	0.30
				BD 4 minutes bead beating at 6V, no versus 0.4x bead/sample AMPure	0.80
				BD 4 minutes bead beating, no versus 0.8x bead/sample AMPure	0.50
				BD 4 minutes bead beating, no versus 2 rounds of 0.4x bead/sample AMPure	0.54
				BD 4 minutes bead beating, 0.8x bead/sample AMPure versus 0.4x bead/sample AMPure	0.49
				BD 4 minutes bead beating, 1 versus 2 rounds of 0.4x bead/sample AMPure	0.50
				BD 4 minutes bead beating, 1 round 0.8x bead/sample AMPure versus 2 rounds of 0.4x bead/sample AMPure	0.91
				BD 4 minutes bead beating at 6V, 1 round 0.4x bead/sample AMPure with DNAexpress purification versus same method with QuickDNA purification	0.74

Table S1: Summary of statistical comparisons between methods. In each case, the normal distribution and equality of variances were tested using the Shapiro-Wilk and Bartlett's tests, respectively.

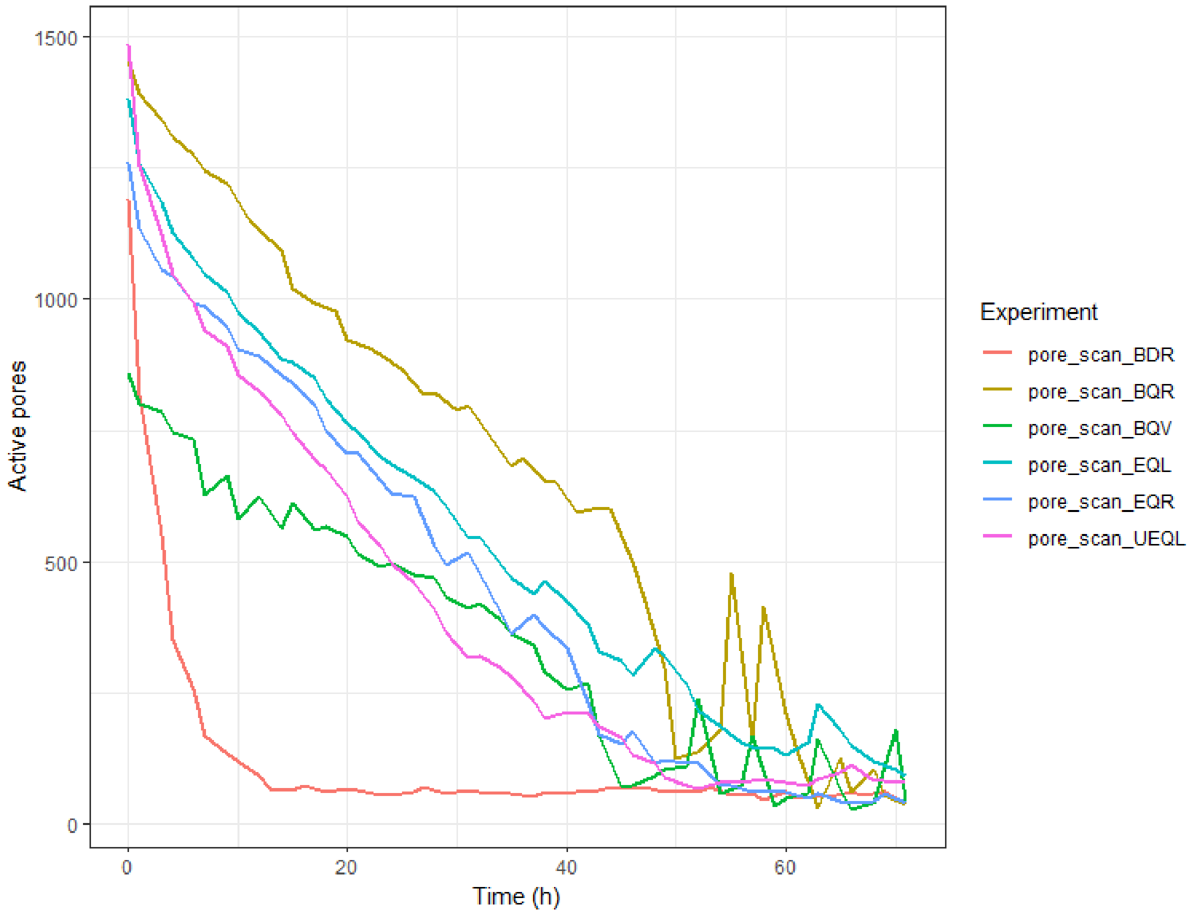

Figure S1: Decay of active nanopores during the various experiments. UEQL: Unspiked – Enzymatic Lysis – Quick-DNA magbead HMW– Ligation sequencing. EQL: Enzymatic Lysis – Quick-DNA magbead HMW – Ligation sequencing. EQR: Enzymatic Lysis – Quick-DNA magbead HMW – Rapid sequencing. BDR: Bead-beating – DNAexpress – Rapid sequencing. BQR: Bead-beating – Quick-DNA HMW magbead – Rapid sequencing. BQV: Bead-beating – Quick-DNA HMW magbead – Voltrax sequencing.

Species	Relative abundance in DMC (%)	UEQL*	BQR	BQV	BDR	EQL	EQR
Akkermansia muciniphila	1.45	5.57e-02	1.93	1.54	0.92	2.25	1.22
Allobacillus halotolerans	2.12	2.06e-04	0.49	0.64	0.47	1.20	1.54
Bacteroides fragilis	13.58	15.34	17.57	20.35	10.60	16.43	21.53
Bifidobacterium adolescentis	5.82	2.37e-02	1.08	1.13	2.18	2.81e-02	1.89e-02
Candida albicans	0.74	63.01	4.06	2.22	10.99	1.26	1.44
Clostridioides difficile	1.45	0.14	3.38	1.88	0.54	1.56	1.26
Clostridium perfringens	1.00e-04	0.34	0.22	0.18	0.33	0.19	0.21
Enterococcus faecalis	1.00e-03	6.52e-02	1.30e-02	2.09e-03	5.32	0.24	0.17
Escherichia coli	13.56	6.24	21.34	17.66	12.43	18.69	16.99
Faecalibacterium prausnitzii	13.55	0.39	7.38	7.60	5.92	17.1	3.73
Lactobacillus fermentum	5.83	0.10	0.48	0.50	1.82	5.91	4.83e-02
Prevotella corporis	2.82	5.84	8.51	9.24	4.52	7.82	10.05
Roseburia hominis	13.55	0.13	4.33	6.18	6.50	5.02	6.56
Saccharomyces cerevisiae	0.67	2.23	3.80	0.56	19.46	0.98	0.80
Salmonella enterica	0.01	5.64e-05	1.00	0.57	3.39	2.10e-02	0.17
Veillonella rogosae	13.58	6.09	15.06	23.01	10.1	17.01	28.56
Fusobacterium nucleatum	5.82	NA	8.39	4.94	1.37	3.01	2.31
Imtechella halotolerans	2.36	NA	0.94	1.73	0.63	1.28	3.48
Methanobrevibacter smithii	0.10	NA	4.81e-02	6.72e-02	2.49	2.71e-02	4.60e-02

Table S2: Theoretical abundance in the spike-in defined mock community (DMC), and the observed relative abundances in terms of the proportion of mapped bases for each experiment. UEQL: Unspiked – Enzymatic Lysis – Quick-DNA magbead HMW– Ligation sequencing. EQL: Enzymatic Lysis – Quick-DNA magbead HMW – Ligation sequencing. EQR: Enzymatic Lysis – Quick-DNA magbead HMW – Rapid sequencing. BDR: Bead-beating – DNAexpress – Rapid sequencing. BQR: Bead-beating – Quick-DNA HMW magbead – Rapid sequencing. BQV: Bead-beating – Quick-DNA HMW magbead – Voltrax sequencing. *UEQL was not spiked with the DMC. NA: No reads mapped to this species.

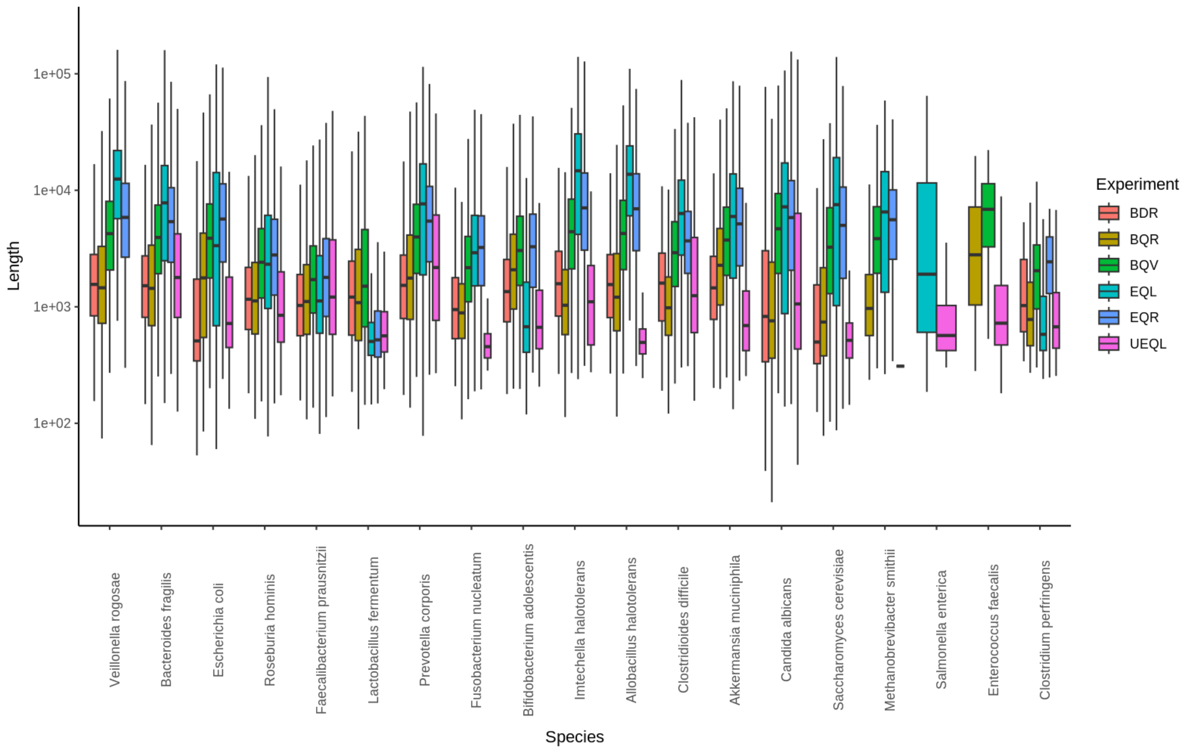

Figure S2: Read length distribution for each species of the spike-in DMC, by experiment. UEQL: Unspiked – Enzymatic Lysis – Quick-DNA magbead HMW– Ligation sequencing. EQL: Enzymatic Lysis – Quick-DNA magbead HMW – Ligation sequencing. EQR: Enzymatic Lysis – Quick-DNA magbead HMW – Rapid sequencing. BDR: Bead-beating – DNAexpress – Rapid sequencing. BQR: Bead-beating – Quick-DNA HMW magbead – Rapid sequencing. BQV: Bead-beating – Quick-DNA HMW magbead – Voltrax sequencing. UEQL was not spiked with the DMC.

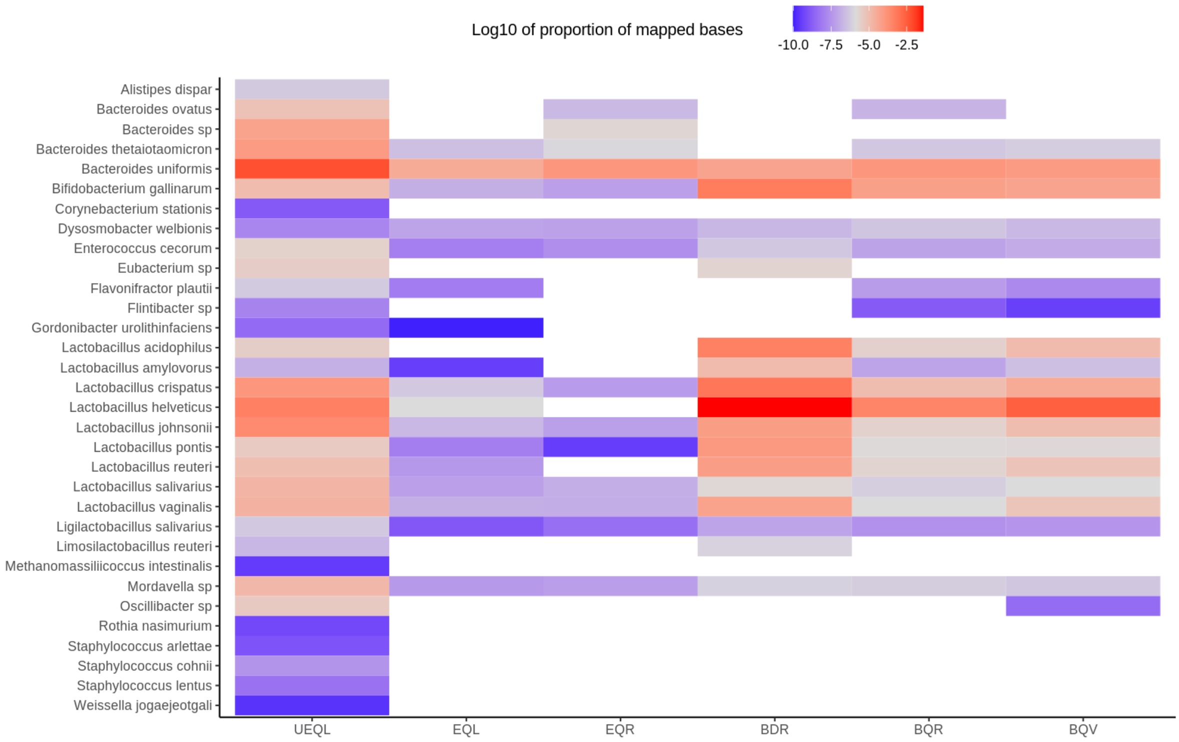

Figure S3: Species detected in the fecal background (UEQL) with query identity > 80% and template coverage > 5%, excluding the spike-in defined mock community members when using a broad taxonomic database. White tiles indicate that the species was not detected. UEQL: Unspiked – Enzymatic Lysis – Quick-DNA magbead HMW– Ligation sequencing. EQL: Enzymatic Lysis – Quick-DNA magbead HMW – Ligation sequencing. EQR: Enzymatic Lysis – Quick-DNA magbead HMW – Rapid sequencing. BDR: Bead-beating – DNAexpress – Rapid sequencing. BQR: Bead-beating – Quick-DNA HMW magbead – Rapid sequencing. BQV: Bead-beating – Quick-DNA HMW magbead – Voltrax sequencing. UEQL was not spiked with the DMC.

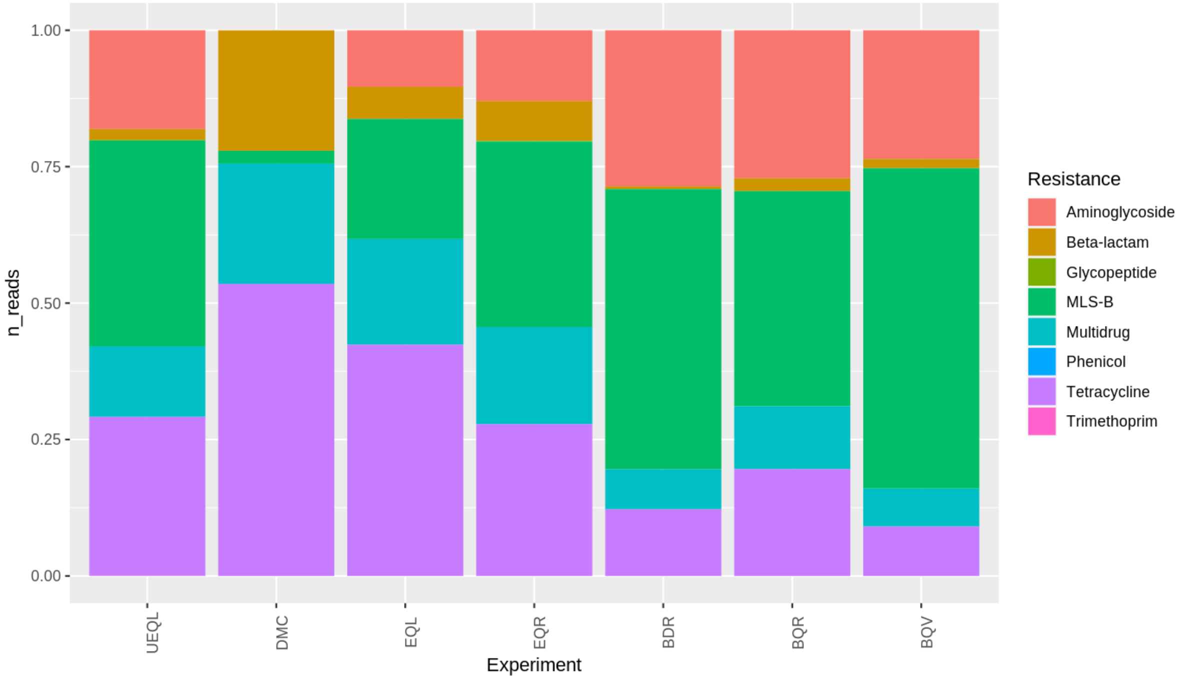

Figure S4: Normalized distribution of antibiotic resistance classes. MLS-B: Macrolide-Lincosamide-Streptogramin-B resistance. UEQL: fecal background consisting of Unspiked fecal sample – Enzymatic Lysis – Quick-DNA magbead HMW– Ligation sequencing. EQL: Enzymatic Lysis – Quick-DNA magbead HMW – Ligation sequencing. EQR: Enzymatic Lysis – Quick-DNA magbead HMW – Rapid sequencing. BDR: Bead-beating – DNAexpress – Rapid sequencing. BQR: Bead-beating – Quick-DNA HMW magbead – Rapid sequencing. BQV: Bead-beating – Quick-DNA HMW magbead – Voltrax sequencing. UEQL was not spiked with the reference community. DMC: abundance of resistance gene classes in the spike-in defined mock community.

Reference sequence	Template ARG	Template length	Template Identity	Template Coverage	Query Identity	Query Coverage	Depth	Q value	P value
R. hominis (Defined mock community)	*blaAST-1*	933	0.00	0.00	0.00	0.00	0.00	23.96	9.8e-07
	mcr-4.1	1626	0.00	0.00	0.00	0.00	0.00	16.93	3.9e-05
	tlr(C)	1647	0.00	0.00	0.00	0.00	0.00	18.93	1.4e-05
R. hominis (NZ_LR699011.1)	*ant(6)-Ia*	909	0.00	0.00	0.00	0.00	0.00	491.40	1.0e-26
	aph(3')-IIIa	795	99.87	100.00	99.87	100.00	1.00	786.97	1.0e-26
	mcr-4.1	1626	0.00	0.00	0.00	0.00	0.00	12.23	4.7e-04
	tlr(C)	1647	0.00	0.00	0.00	0.00	0.00	14.11	1.7e-04
	tet(W)	1920	99.90	100.00	99.90	100.00	1.00	1909.35	1.0e-26

Table S3: Antibiotic resistance gene (ARG) detected by KMA and the ResFinder database, in *Roseburia hominis* reference genomes obtained from the DMC or from NCBI refseq.

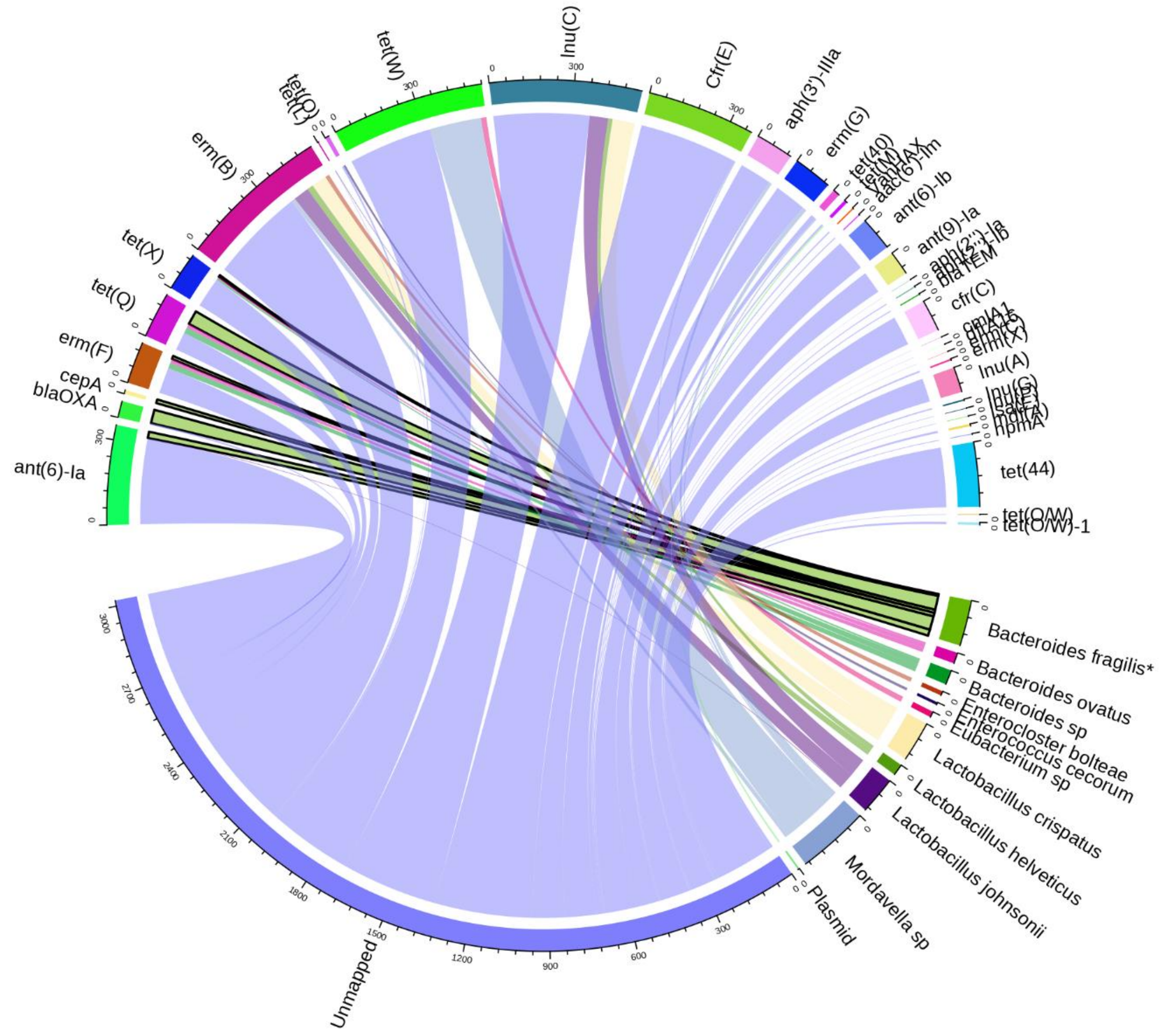

Figure S5: Antimicrobial resistance gene – host links for experiment UEQL: Unspiked – Enzymatic Lysis – Quick-DNA magbead HMW– Ligation sequencing. Read counts are indicated by the circular scale. Connections from ARG to spike-in DMC members are highlighted in bold. *: Members of the spiked-in DMC.

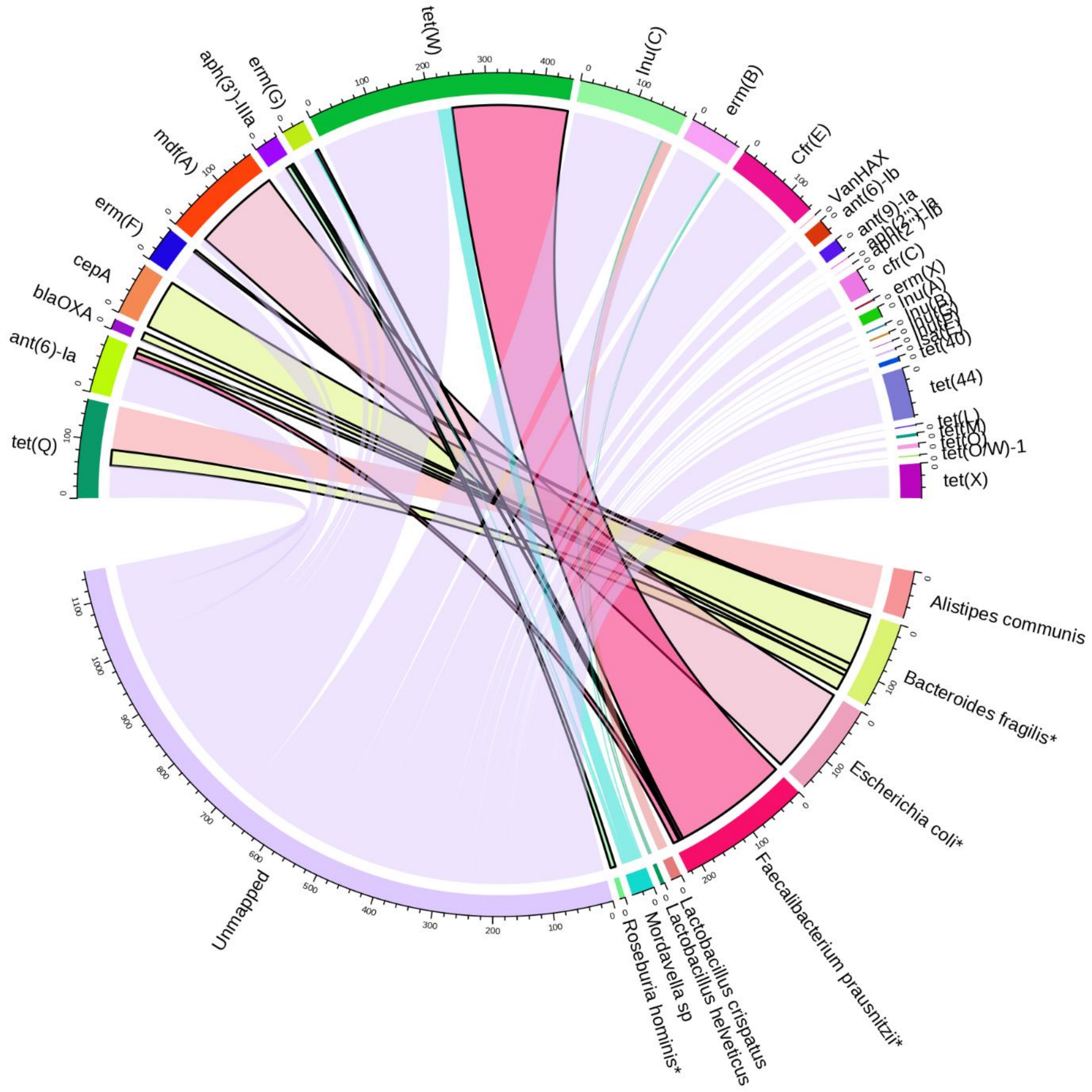

Figure S6: Antimicrobial resistance gene – host links for experiment EQL: Enzymatic Lysis – Quick-DNA magbead HMW – Ligation sequencing. Read counts are indicated by the circular scale. Connections from ARG to spike-in DMC members are highlighted in bold. *: Members of the spiked-in DMC.

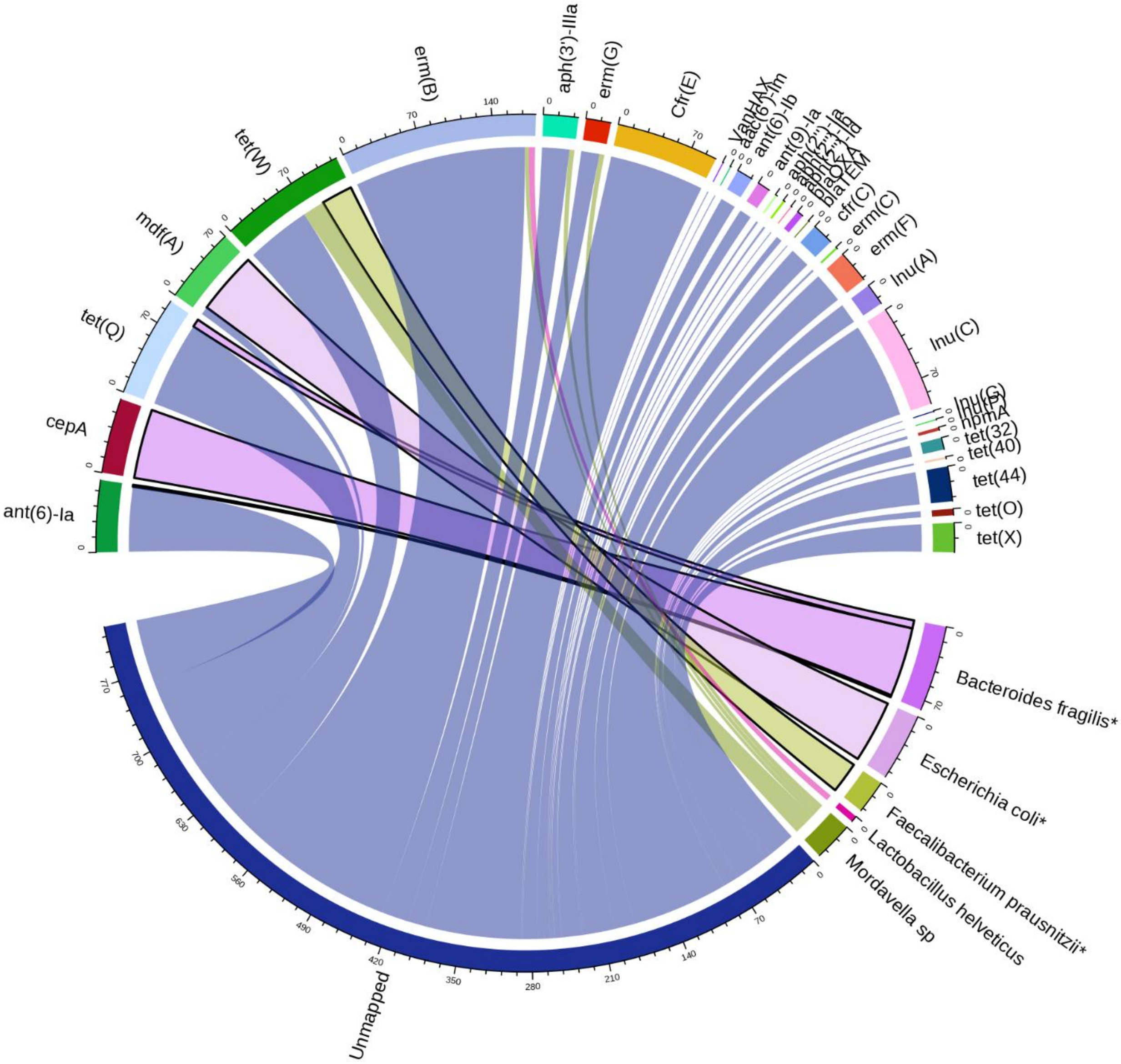

Figure S7: Antimicrobial resistance gene – host links for experiment EQR: Enzymatic Lysis – Quick-DNA magbead HMW – Rapid sequencing. Read counts are indicated by the circular scale. Connections from ARG to spike-in DMC members are highlighted in bold. *: Members of the spiked-in DMC.

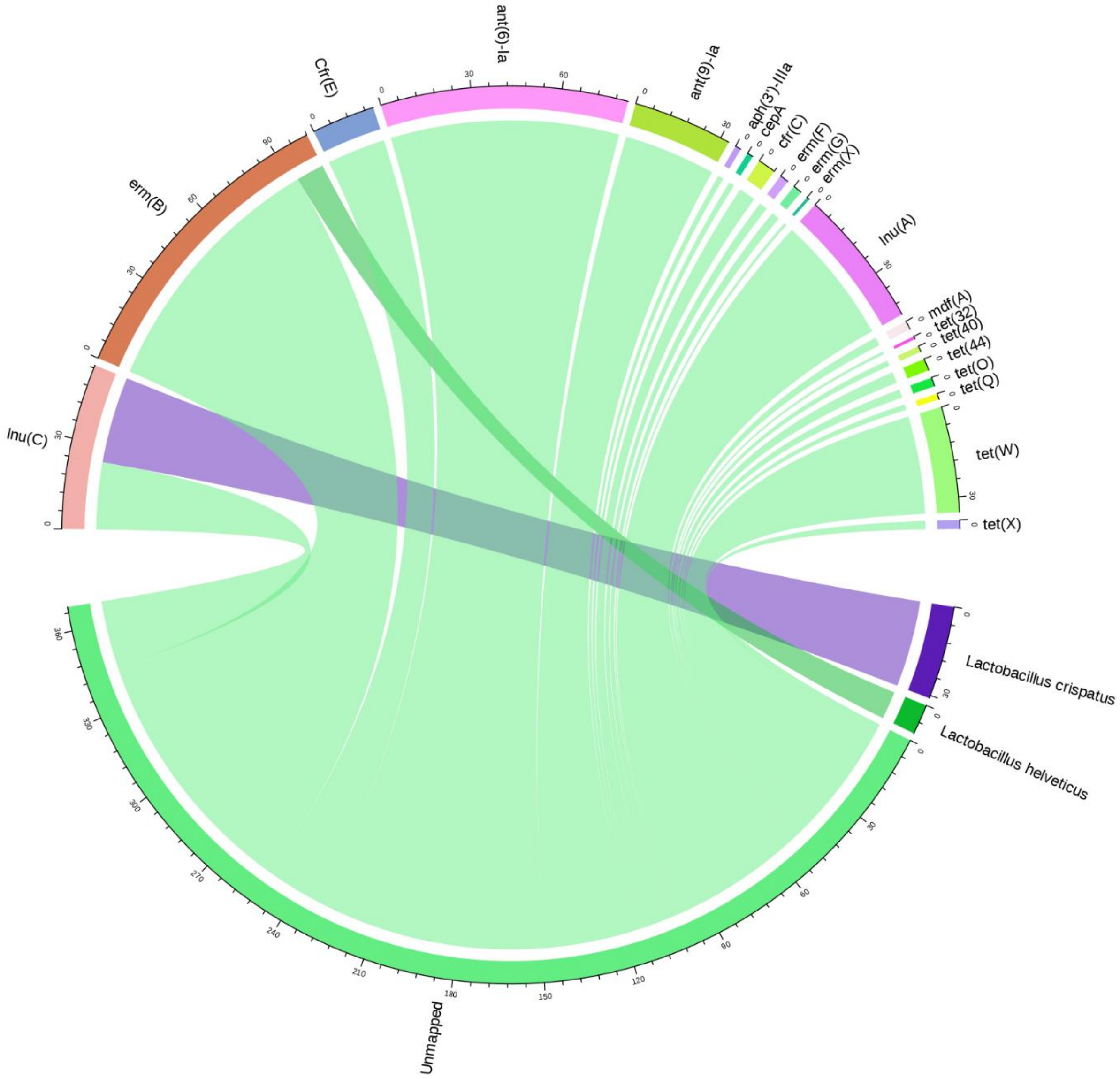

Figure S8: Antimicrobial resistance gene – host links for experiment BDR: Bead-beating – DNAexpress – Rapid sequencing. Read counts are indicated by the circular scale. Connections from ARG to spike-in DMC members are highlighted in bold. *: Members of the spiked-in DMC.

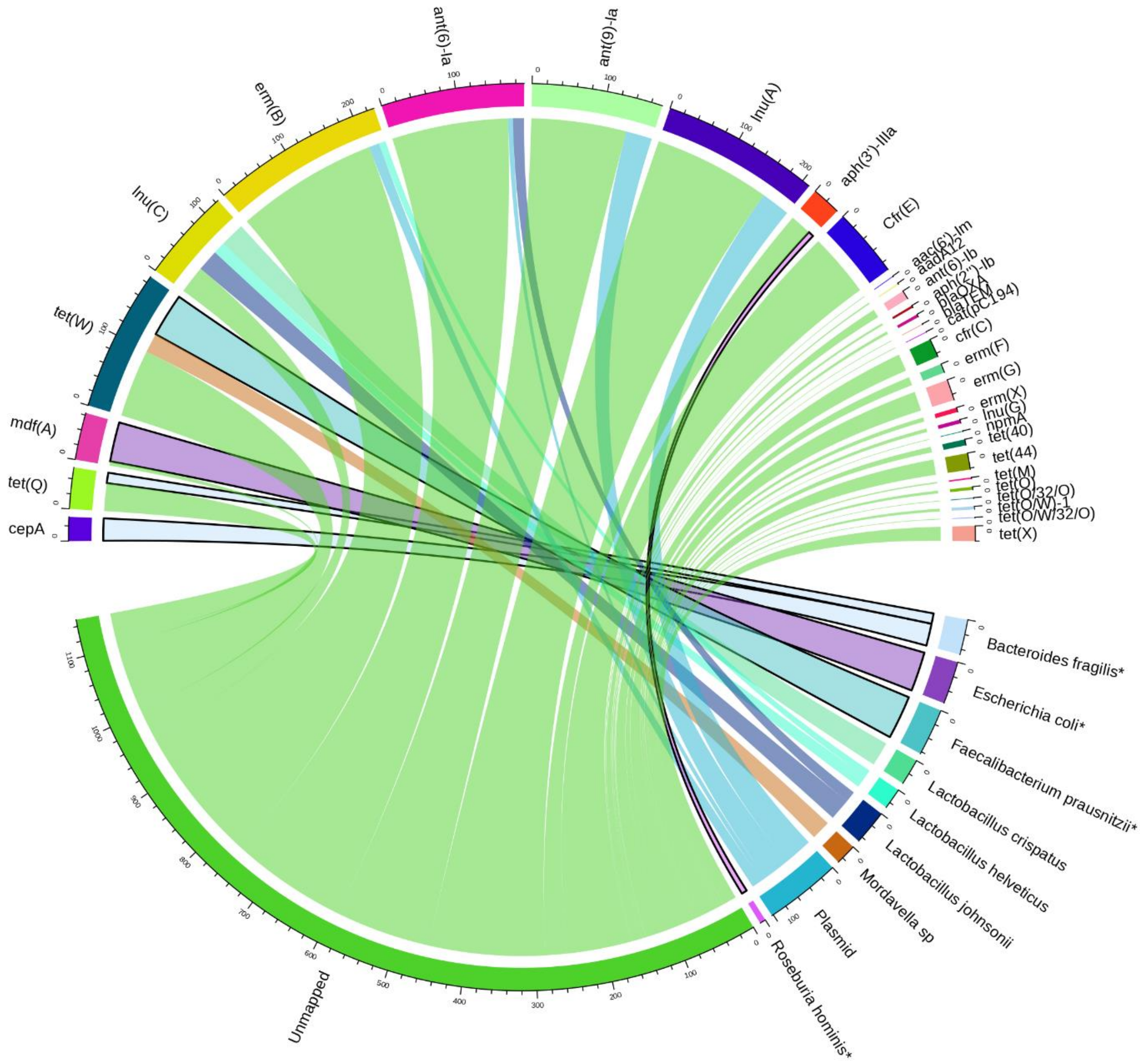

Figure S9: Antimicrobial resistance gene – host links for experiment BQR: Bead-beating – Quick-DNA HMW magbead – Rapid sequencing. Read counts are indicated by the circular scale. Connections from ARG to spike-in DMC members are highlighted in bold. *: Members of the spiked-in DMC.
